# Role of Intra- and Extracellular Lipid Signals in Cancer Stemness and Potential Therapeutic Strategy

**DOI:** 10.3389/fphar.2021.730751

**Published:** 2021-09-15

**Authors:** Jianming Hu, Leyi Zhang, Wuzhen Chen, Lesang Shen, Jingxin Jiang, Shanshan Sun, Zhigang Chen

**Affiliations:** ^1^ Department of Breast Surgery (Surgical Oncology), Second Affiliated Hospital, Zhejiang University School of Medicine, Hangzhou, China; ^2^ Key Laboratory of Tumor Microenvironment and Immune Therapy of Zhejiang Province, Hangzhou, China

**Keywords:** lipid metabolism, cancer stem cell, tumor environment, ferroptosis, therapeutic target

## Abstract

Accumulating evidence showed that cancer stem cells (CSCs) play significant roles in cancer initiation, resistance to therapy, recurrence and metastasis. Cancer stem cells possess the ability of self-renewal and can initiate tumor growth and avoid lethal factors through flexible metabolic reprogramming. Abnormal lipid metabolism has been reported to be involved in the cancer stemness and promote the development of cancer. Lipid metabolism includes lipid uptake, lipolysis, fatty acid oxidation, *de novo* lipogenesis, and lipid desaturation. Abnormal lipid metabolism leads to ferroptosis of CSCs. In this review, we comprehensively summarized the role of intra- and extracellular lipid signals in cancer stemness, and explored the feasibility of using lipid metabolism-related treatment strategies for future cancer.

## Introduction

Cancer stem cells (CSCs) have been found in many common cancer types, including breast, colorectal, brain cancer and leukemia. However, more and more people have realized that not all cancer types adhere to the CSC model (Batlle and Clevers, 2017). For example, no CSCs were found in melanoma (Quintana et al., 2008). Generally, CSCs show a high potential of plasticity, which can transform from a quiescent state to a proliferative state and/or commit to a differentiated state when activated. Cancer stem cells, including tumor-initiating cells (TICs), are a small subgroup of cancer cells exhibiting self-renewal and tumor-initiating properties, which account for cancer initiation, metastasis, resistance to therapy, recurrence, and poor prognosis (Libby et al., 2018). Therefore, targeting CSC is a promising therapeutic strategy. However, there is a lack of targeted drugs because CSC has strong plasticity. In some cases, CSCs appear to be derived from tissue stem cells that gain oncogenic transformation (Batlle and Clevers, 2017), while in other cases, tumor cells possess stem-cell traits of becoming CSCs (Prasetyanti and Medema, 2017).

Accumulating evidence showed that tumor cells acquired stem-cell traits through metabolic reprogramming. Cancer stem cells have been reported to be metabolically different from regular cancer cells. According to the report, glioma stem cells are different from their progeny and rely mainly on oxidative phosphorylation (Vlashi et al., 2011). As mentioned earlier, CSCs can either reside in a quiescent state or proliferate vigorously. The lifecycle of stem cells is a metabolism-dependent process comprising of maintenance and acquisition of stemness to lineage commitment and specification (Folmes and Terzic, 2016). They have an oxidative phenotype when they are quiescent, and switch to a combined glycolytic/oxidative metabolic program when they are forced to proliferate (Peiris-Pagès et al., 2016). Therefore, it is emerging that there is no universal metabolic pattern to distinguish CSCs from non-CSCs. Cancer stem cells and non-CSCs preferentially use glycolysis or oxidative phosphorylation, depending on the tumor type and research model used (Batlle and Clevers, 2017).

In addition to studies focusing on glucose metabolism of cancers, the other studies also have indicated that CSCs extremely rely on the lipid metabolism. Cholesterol and fatty acids (FA) are not only the important components of animal cell membranes but also precursors for a wide variety of biological molecules. Due to the potential toxic effects of excessive accumulation of cholesterol and fatty acids on individual cells and the whole animal, their expression must be strictly regulated (DeBose-Boyd, 2018). Lipid metabolism has been regarded as the key factors for the correct function of pathways involved in CSC fate decision and characteristics of CSC like chemotherapy evasion (Ye et al., 2016; Mancini et al., 2018). The enhanced lipid metabolism is essential for the survival, growth, and oncogenicity of CSCs (Libby et al., 2018; Pan et al., 2020). Fatty acids serve as another fuel pathway for CSCs. Increased levels of lipids and fatty acid oxidation (FAO)-related genes have been observed in CSCs. The elevated FAO can maintain CSCs self-renewal by modulating lipid and membrane synthesis, quenching ROS through NADPH production, and promoting chemoresistance (Daniel et al., 2021). A lipogenic switch is observed in CSCs, which facilitate the production of monounsaturated lipids that are less susceptible to lipid peroxidation, thus restricting the detrimental effect of ROS and contributing to cancer progression and metastasis (Vazquez-Martin et al., 2013; Daniel et al., 2021). Researchers have tried to invent alternative approaches targeting key regulators in CSC lipid metabolism, but many of them still face challenges.

Cancer stem cells possess indefinite self-renewal ability to initiate and maintain tumor growth, and they can avoid lethal factors through their flexible metabolic reprogramming. Therefore, targeting CSCs is of great significance for reducing the risk of resistance to therapy, recurrence, and metastasis. Lipid metabolism reprogramming has been widely seen in CSCs, but the extent to which changes affect CSCs remains a mystery and the underlying mechanisms that regulate this metabolic plasticity need to be further elucidated. In this review, we summarized the reprogramming of lipid metabolism, including intracellular lipid signals in CSCs, lipid droplets contents, lipid uptake, lipolysis, fatty acid oxidation, lipid desaturation, lipid peroxidation, and the influence of lipid signals in components of the tumor microenvironment (TME) on CSCs, and explore the potential lipid metabolism-related targets for cancer therapy.

### Intra Lipid Signals Alterations IN CSCs

#### Lipid Synthesis

In normal conditions, *de novo* lipogenesis is strictly regulated, and the excess carbohydrates are converted into lipids through a series of reactions. It mainly happens in specific locations, such as liver or adipose tissue (Ameer et al., 2014). However, the characteristics of cancer cells, including vigorous metabolism, rapid proliferation, and production of ATP through glycolysis, form a TME that is nutritionally deficient, acidified, and hypoxic (Arneth, 2019; Vitale et al., 2019; Bader et al., 2020). Therefore, compared with normal cells, cancer cells are highly dependent on *de novo* lipogenesis for survival and growth. It is pointed out that *de novo* lipogenesis produced over 90% of the lipids stored in lipid droplets (LDs) in cancer cells (Menendez and Lupu, 2007). In addition, even when exogenous fatty acids are sufficient, *de novo* lipogenesis is still upregulated (Mashima et al., 2009). The conversion of lipid acquisition from lipid intake to *de novo* lipogenesis had a protective effect on CSCs. It supported the survival of CSCs from both endogenous and exogenous injuries and enhanced the resistance to radiotherapy and chemotherapy through transformation of membrane properties (Rysman et al., 2010).

**FIGURE 1:**
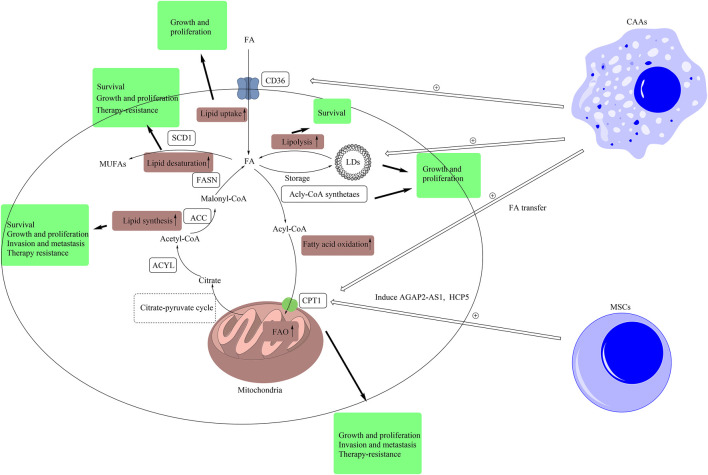
Lipid signals alteration in CSCs and TME. Intracellular lipid signals comprise of lipid uptake, lipolysis, fatty acid oxidation, lipid synthesis, lipid desaturation, and lipid peroxidation. In this figure, we have briefly exhibited the pathways of lipid uptake, lipolysis, fatty acid oxidation, lipid synthesis, and lipid desaturation. The metabolic products of other forms of metabolism, such as citrate produced in glucose metabolism, are transported out of mitochondria by citrate-pyruvate cycle. Then, citrate is converted to malonyl-CoA by the catalysis of ACLY and ACC. Malonyl-CoA is used by FASN to synthesize FAs. Redundant FAs result from *de novo* synthesis and uptake by CD36 are stored in LDs, and LDs provide FAs by lipolysis. Fatty acids can be converted into acyl-CoA, and the latter can be transported into mitochondria by CPT1 for fatty acid β-oxidation. Besides, SCD1 can catalyze FAs into MUFAs. In CSCs, these pathways are abnormally upregulated. The pathway of lipid synthesis is closely related with the stemness features, including cell growth and proliferation, invasion and metastasis, and resistance to therapy, in CSCs. The inhibition of lipid synthesis induces the death of CSCs. The upregulation of lipid desaturation supports the growth, proliferation, and resistance to therapy in CSCs. Inhibiting SCD1 improves the sensibility of CSCs to ferroptosis. The higher level of LDs promotes the growth and proliferation of CSCs. Blockage of lipolysis leads to the death of CSCs. The upregulation of lipid uptake supports the growth and proliferation of CSCs. The upregulation of FAO has a positive effect on the growth, proliferation, invasion, metastasis, and resistance to therapy in CSCs. Extracellular lipid signals in TME also support the stemness of CSCs. Mesenchymal stem cells induce the expression of AGAP2-AS1 and HCP5 to elevate FAO in CSCs to support the stemness of CSCs. Besides, CAAs improve the levels of LDs and the expression of CPT1 and CD36 by secreting cell factors or lipid transfer to support the stemness of CSCs. (ACLY, ATP citrate lyase; ACC, acetyl-CoA carboxylase; CAA, cancer-associated adipocytes; FA, fatty acid; FAO, fatty acid oxidation; FASN, fatty acid synthase; CD36, cluster of differentiation 36; LD, lipid droplet; CPT1, carnitine palmitoyltransferase-1; MUFA, monounsaturated fatty acid; MSC, mesenchymal stem cell; SCD1, stearoyl-CoA desaturase 1; TME, tumor microenvironment).

**FIGURE 2:**
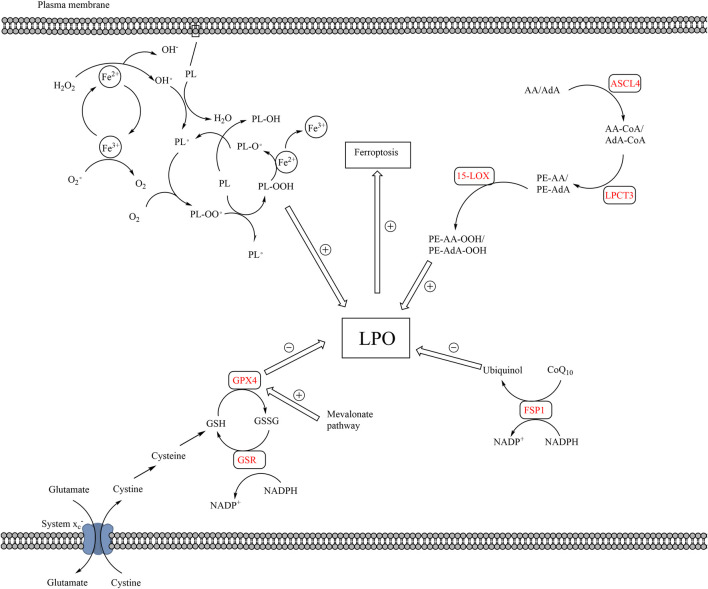
The pathways of lipid peroxidation and ferroptosis. The production of LPO mainly results from auto-oxidation of lipids and enzymatic lipid peroxidation. Auto-oxidation of lipid is a radical reaction, which means the upstream reaction can induce the downstream reaction. PL is converted to PL• by ROS, which is the production of Fe^2+^ and H_2_O_2_. PL• reacts with O_2_ to form PL-OO•, which further reacts with a new PL to form PL-OOH and a new PL•, proceeding downstream radical reaction. Enzymatic lipid peroxidation mainly takes place in AA or AdA. AA and AdA can be converted into PE-AA-OOH and PE-AdA-OOH by the catalysis of ACSL4, LPCAT3, and 15-LOX. The elimination of LPO mainly relies on GPX4 and FSP1. Cystine is transported into cells *via* system x_c_^−^, which is the raw material for the production of GSH. GPX4 converts GSH to GSSH and reduces LPO in the meantime. Besides, FPS1 converts CoQ_10_ to ubiquinol, which reduces LPO. The accumulation of LPO, caused by excessive production or blockage of elimination of LPO, leads to ferroptosis. (15-LOX, 15-lipoxygenases; AA, arachidonoyl; ACSL4, acyl-CoA synthetase long-chain family member 4; AdA, adrenoyl; CoQ_10_, coenzyme Q_10_; FSP1, ferroptosis suppressor protein 1; LPO, lethal lipid peroxides; LPCAT3, lysophosphatidylcholine acyltransferase 3; PL, phospholipids; PL•, phospholipid radical; GPX4, glutathione peroxidase 4; GSH, glutathione; GSSG, glutathione disulfide; ROS, reactive oxygen species).

Accumulating studies have shown that the key enzymes in the *de novo* lipogenesis, including fatty acid synthase (FASN), acetyl-CoA carboxylase (ACC), ATP-citrate lyase (ACLY) (Figure 1), were abnormally upregulated in CSCs, leading to the upregulation of *de novo* lipogenesis (Gopal et al., 1963; Santos and Schulze, 2012; Ameer et al., 2014; Bergroth et al., 2016). For example, in breast cancer cells, the enhanced expression of ACLY was related to the upregulation of snail proteins, which triggered tumorigenesis and enhanced cancer stemness (Hanai et al., 2013). These upregulated key enzymes could promote tumor growth in many cancer types, such as breast, prostate and non-small cell lung cancer (Al-Bahlani et al., 2017; Lee et al., 2017; Singh et al., 2018; Mouhid et al., 2019; Lounis et al., 2020; Yang D. et al., 2020; Simeone et al., 2021). In the meantime, studies have proved that drugs that inhibited the expression of these key enzymes, such as resveratrol and bakuchiol, could restrain the stemness of CSCs and achieve a certain curative effect (Table 1; Figure 2). Pandey et al. used resveratrol, a kind of hypolipidemic drug, successfully restrained the growth of breast CSCs, because resveratrol could restrain the *de novo* lipogenesis by inhibiting the expression of FASN and inducing apoptosis of CSCs (Pandey et al., 2011). Similarly, bakuchiol could target breast CSCs by modulating the expression levels of Notch3 and FASN in the zebrafish embryos model (Li L. et al., 2017).

**TABLE 1:** Inhibitors of lipid metabolism involved in CSCs.

Metabolism types	Drug	Target	Cancer types	Function	Refs
Lipid uptake	CD36 antibody	CD36	Oral carcinomas	Blocking metastatic potential of CD36^+^ oral carcinoma in a mouse model	Pascual et al. (2017)
			Mantle Cell lymphoma CSCs	Inhibiting the resistance to bortezomib	Luanpitpong et al. (2019)
	MTN	CD36	Glioblastoma CSCs	Inhibiting the capabilities of self-renewal and tumor initiation	Hale et al. (2014)
Lipid synthesis	Resveratrol	FASN	Breast CSCs	Inhibiting the expression of FASN and result in CSCs apoptosis	Pandey et al. (2011)
			Glioblastoma CSCs	Affecting the Wnt signaling and restraining the survival and motility of CSCs	Cilibrasi et al. (2017)
	
	Microrna-127 pro-drug	FASN	Triple negative breast cancer	Retraining survival and growth of CSCs and the resistance to chemotherapy	Umeh-Garcia et al. (2020)
	Cerulenin	FASN	Pancreatic CSCs	Inhibiting FASN and restraining the aggressiveness of pancreatic CSCs	Brandi et al. (2017)
			Glioma CSCs	Restraining the capabilities of proliferation and migration	Yasumoto et al. (2016)
	Bakuchiol	FASN	Breast CSCs	Inhibiting metastasis and inducing apoptosis	Tirinato et al. (2017)
	C75	FASN	Luminal-B breast CSCs	Inhibiting the endocrine resistance of CSCs	(Menendez et al., 2020; Menendez et al., 2021)
	G28	FASN	Triple negative breast cancer	Inhibiting the mammosphere-formation capacity of CSCs	Giró-Perafita et al. (2019)
	Orlistat	FASN	Mutated EGFR non-small cell lung cancer	Inducing the EGFR ubiquitination and making CSCs re-sensitive to TKI	Ali et al. (2018)
	Soraphen A	ACC	Breast CSCs	Inhibiting mammosphere formation	Vancampfort et al. (2014)
	TOF A	ACC	Breast CSCs	Downregulating the expression of ACC and the load of lds, and suppressing the stemness of breast cancer	Hershey et al. (2019)
	Atorvastatin	Mevalonate pathway	Pancreatic ductal CSCs	Inhibiting the growth of CSCs	Brandi et al. (2017)
	Metformin	Mevalonate pathway	Colorectal CSCs	Inhibiting the survival of CSCs	Seo et al. (2020)
	Lipophilic statins such as atorvastatin, lovastatin, and simvastatin	Mevalonate pathway	Breast CSCs	Inhibiting the captivity of EMT in CSCs	Koohestanimobarhan et al. (2018)
	Simvastatin	Mevalonate pathway	Ovarian CSCs	Inhibiting the plasticity of CSCs and metastasis	Kato et al. (2018)
	Pyrvinium pamoate	Lipid synthesis	Triple-negative breast cancer	Impairing the anabolic flux from glucose to cholesterol and fatty acids	Dattilo et al. (2020)
FAO	Etomoxir	CPT1	Acute myeloid leukemia CSCs	Inhibiting the survival of CSCs	Samudio et al. (2010)
		CPT1	Gastric CSCs	Inhibiting the stemness and chemotherapy of CSCs	He et al. (2019)
	ST1326	CPT1	Lymphoma	Induction of lipotoxicity	(Pacilli et al., 2013; Ricciardi et al., 2015)
			Acute myeloid leukemia		
Lipolysis	TOFA	Not known	Ovarian CSCs	Inhibiting the EMT of CSCs	Pouyafar et al. (2019)
Lipid desaturation	MF-438	SCD1	Ovarian CSCs	Inducing ferroptosis	Wang et al. (2018b)
			Lung CSCs	Modulation of the resistance to therapy in CSCs	Pisanu et al. (2017)
	SSI-4	SCD1	Hepatocellular CSCs	Modulation of the resistance to therapy in CSCs	Wang et al. (2018b)
	A939572	SCD1	Ovarian CSCs	Inducing to ferroptosis	Wang et al. (2018b)
		SCD1	Hepatocellular CSCs	Suppressing the captivities of self-renewal and metastasis and the resistance to sorafenib	Li et al. (2017a)
	BetA	SCD1	Colorectal cancer	Inducing rapid cell death in all colon CSCs	Potze et al. (2016)
	Cay10566	SCD1	Ovarian CSCs	Inducing to ferroptosis and inhibiting the proliferation of CSCs	Wang et al. (2018b)
		SCD1	Ovarian CSCs	Inhibiting the growth of ovarian CSCs	Kagan et al. (2017)
		SCD1	Glioma CSCs	Inhibiting the growth of glioma CSCs	Pinkham et al. (2019)
	T-3764518	SCD1	Colorectal cancer	Activating endoplasmic reticulum stress responses	Zhang et al. (2014)
Lipid peroxidation	Knockdown of Frizzled-7	GPX4	Platinum-tolerant cancer cell line	Reversing the resistance to therapy and suppressing stemness	Wang et al. (2021)
	RSL3	GPX4	Ovarian adenocarcinoma cells	Reversing multidrug resistance to chemotherapy	Gao et al. (2019)
	siRNA	GPX4	Pancreatic CSCs	Knockdown of GPX4 and suppressing stemness	Peng et al. (2019)
	duplexes					
	Salinomycin	Iron	Breast CSCs	Inducing ferroptosis	Mai et al. (2017)
TME	Frax-NPCGKRK	CAFs	Pancreatic CSCs	Inhibiting the dense stroma barrier	Pei et al. (2019)

CSCs, cancer stem cells; MTN, 2-methylthio-1,4-naphthoquinone; FASN, fatty acid synthase; ACC, acetyl-CoA carboxylase; CPT1, carnitine palmitoyltransferase-1; SCD1, stearoyl-CoA desaturase 1; GPX4, glutathione peroxidase 4; TME, tumor microenvironment; CAFs, cancer-associated fibroblasts; EGFR, epidermal growth factor receptor; TKI, tyrosine kinase inhibitor.

In addition to fatty acid synthesis, the mevalonate pathway, which is the pathway of cholesterol synthesis, also plays an important role in the modulation of the stemness of CSCs. The cellular cholesterol is closely related to the stemness features of CSCs, such as resistance to therapy and vigorous growth. For example, in gallbladder cancer, the depletion of cholesterol made CSCs sensitive to cisplatin (Zhang Y. et al., 2019). The transcription factor, c-MYC, could promote the stemness of CSCs by regulating the mevalonate pathway (Wang et al., 2017). More and more studies have reported that the inhibition of the mevalonate pathway suppressed the stemness of CSCs. A series of inhibitors targeting the key enzymes of the mevalonate pathway have been proven to be effectively suppress the stemness of CSCs and inhibit the progression of tumors. The inhibitors of hydroxy-3-methylglutaryl CoA reductase (HMGCR), including statins (Figure 2), could block the mevalonate pathway by inhibiting this key rate-limiting enzyme, thereby impairing the function and stemness of CSCs (Ginestier et al., 2012; Likus et al., 2016; Mattoli et al., 2016; Vásquez-Bochm et al., 2019). Besides, Walsh et al. found that the downregulation of 3-hydroxy-3-methylglutaryl-CoA synthase 1 (HMGCS1), the mevalonate precursor enzyme, inhibited the function of breast CSCs (Walsh et al., 2020). Squalene epoxidase (SQLE), another rate-limiting enzyme in the mevalonate pathway, also plays an important role in the modulation of stemness. It has been proved to be related to the generation of CSCs and the initiation of metastasis by inducing epithelial-mesenchymal transition (EMT) of aggressive colorectal cancer cells. The reduction of SQLE promoted the progression of aggressive colorectal cancer by regulating oncogenic pathway and tumor suppressor pathway (Jun et al., 2021). The lack of SQLE led to the blockage of cholesterol synthesis and the accumulation of upstream metabolite squalene. The accumulation of squalene protected ALK^+^ anaplastic large cell lymphoma cells from oxidative cell death, which provided a survival advantage under conditions of oxidative stress and in tumor xenografts (Garcia-Bermudez et al., 2019). Besides, metformin (Figure 2) could restrain the growth of colorectal CSCs and reduce the number of CSCs by suppressing the mevalonate pathway (Seo et al., 2020). The combined inhibition of FASN and mevalonate pathway had a stronger anti-proliferative effect on pancreatic CSCs than parental cells, which exhibited lower metabolic activity (Brandi et al., 2017).

In summary, due to the deficiency of exogenous lipids in the tumor microenvironment, cancer cells are highly dependent on *de novo* lipogenesis to gain lipids (Yi et al., 2018). *De novo* lipogenesis involves a complicated network of pathways, which means there are plentiful potential targets. The modulation of *de novo* lipogenesis has great potential in the domain of cancer treatment.

#### Lipid Uptake

Compared with normal cells, cancer cells have vigorous metabolism, which requires cancer cells to take up more lipids. Cancer cells uptake lipids through a variety of routes, including CD36/fatty acid translocase, low-density lipoprotein (LDL) mediated endocytosis, and fatty acid transport proteins (Snaebjornsson et al., 2020) (Figure 1). To improve lipid uptake, cancer cells upregulate cell surface receptors, such as CD36 (Koizume and Miyagi, 2016), which is the focus of current research.

CD36 is a transmembrane glycoprotein that mediates the uptake of hydrophobic molecules, such as FAs and cholesterol (Wang and Li, 2019). CD36 is frequently acquired or amplified in CSCs of many cancer types, which is also associated with more aggressive tumor and poorer prognosis (Cancer Genome Atlas Research, 2015; Ladanyi et al., 2018). For example, in glioblastoma, the elevated expression of CD36 promotes the growth of CSCs and helps CSCs to maintain stemness (Hale et al., 2014). Besides, in ovarian CSCs, the expressions of CD36 and other enzymes in lipid metabolism such as ACC, stearoyl-CoA desaturase (SCD), and carnitine palmitoyltransferase 1 (CPT1), were upregulated compared to well-established ovarian cancer cells. The reprogramming of lipid metabolism improved the metabolic plasticity of CSCs, which maintained the stemness of CSCs and provided survival advantages for CSCs. The author pointed out that the recurrence of drug-resistant ovarian cancer was common, which might be mainly caused by the residual CSCs. Targeting lipid uptake might be a promising approach to improve the prognosis of ovarian cancer (Ghoneum et al., 2020). Furthermore, the upregulated expression of CD36 was found to play an important role in the process of metastasis. Pascual et al. found that a subgroup of CSCs with higher CD36 expression made an important contribution to the oral cancer metastasis in mouse models. The upregulated expression of CD36 enhanced the ability of CSCs to initiate metastasis by upregulating the expression of metastasis-related genes. Besides, the CSCs with higher CD36 expression could take up more exogenous lipids, which could be promoted by palmitic acid or a high-fat diet. The expression of genes associated with lipid metabolism, such as lipid β -oxidation, was also upregulated in this subgroup of CSCs, which supported the survival and growth of CSCs at sites of metastasis. The blockage of CD36 could inhibit the metastasis of oral cancer and reduced the size of lymph node metastases. This study indicated that therapies targeting CD36 had great potential to improve the prognosis of cancers by inhibiting metastasis. Clinically, it was reported that the inhibition of CD36 could inhibit the metastasis of melanoma and breast cancer (Pascual et al., 2017) (Table 1; Figure 2).

In summary, the upregulated expression of CD36 plays an important role in the mediation of stemness in CSCs, such as oral, ovarian, and glioblastoma CSCs, and is associated with the cancer metastasis. The blockage of CD36 can effectively inhibit the stemness of CSCs and inhibit the progression of cancers in several studies. Therefore, drugs targeting CD36 have potential to target cancer, and we may improve the prognosis of cancers by reducing the intake of lipids in the diet.

#### Lipid Storage

Lipid droplet is a kind of lipid storage organelles and the hub for lipid metabolic processes (Walther et al., 2017) (Figure 1). Lipid droplets have been proved to play an important role in maintaining cell homeostasis, such as preventing lipotoxicity and protecting against mitochondrial damage during autophagy (Olzmann and Carvalho, 2019). The biogenesis of LDs mainly relies on three lipid pools: extracellular lipid uptake, endogenous *de novo* lipogenesis, and hydrolyzed endogenous structural lipids (Walther et al., 2017). The decomposition of LDs relies on lipolysis or lipophagy (Yi et al., 2018), and the interaction of the biogenesis and decomposition of LDs affects the level of LDs in CSCs (Cruz et al., 2020). Changes in LDs in CSCs have been observed in several cancer types, including colorectal, ovarian cancer and melanoma (Tirinato et al., 2015; Giampietri et al., 2017; Stockwell et al., 2017; Tirinato et al., 2017; Barreno et al., 2019).

Compared with the regular tumor cells, CSCs have more LDs in ovarian and colorectal cancer (Tirinato et al., 2015; Stockwell et al., 2017; Tirinato et al., 2017). For example, Tirinato et al. have proved that the level of LDs was highly elevated in the CSCs of colorectal cancer, which was the sign of CSCs. They observed that the load of intracellular LDs was directly related to the expression level of the colorectal CSCs markers, such as CD133 and Wnt pathway. Compared with the colorectal CSCs with lower content of LDs content, colorectal CSCs with higher LDs content appear to show higher stemness features (Tirinato et al., 2015). Similarly, the enrichment of intracellular LDs was related to the stemness of the BT474 breast cancer cell line (Hershey et al., 2019). The upregulation of LDs enhances the stemness of CSCs, and the downregulation of LDs also impairs the stemness of CSCs. An inhibitor of acetyl-CoA carboxylase-α, 5-(tetradecyloxy)-2-furoic acid, could block endogenous *de novo* FA synthesis and reduce intracellular LDs load. It could significantly inhibit second-generation mammosphere-forming ability of BT474 breast cancer cell line (Hershey et al., 2019).

In addition, it has been reported that elevated LDs can support the survival of CSCs. Based on the Warburg effect, tumor cells tend to produce energy through aerobic glycolysis rather than oxidative phosphorylation (Liberti and Locasale, 2016). Therefore, the upregulated LDs provide an alternative energy source to CSCs when glycolysis is inhibited (Bailey et al., 2015). Besides, as lipid storage organelles, LDs limit the contact and reaction between lipid and reactive oxygen species (ROS) and protect CSCs from ferroptosis (Hershey et al., 2019).

In summary, the accumulated LDs maintain the stemness of CSCs and benefit the survival of CSCs, helping CSCs survive in the situation where glycolysis is restrained and protecting CSCs from peroxidation. Therapies designed to downregulate the LDs load in CSCs have potential to inhibit cancer.

#### Lipolysis

Lipolysis defines the process of lipids breakdown, including the reaction of hydrolysis of triglycerides into glycerol and free fatty acids (Zechner et al., 2012) (Figure 1). The lipolysis in CSCs supports the survival, growth, and resistance to therapy of CSCs. ADP-ribosylation factor 1 (Arf1) is located in the Golgi apparatus and plays an important role in intra-Golgi transport, including the transport of lipolytic enzymes to the surface of LDs to initiate lipolysis. The knockdown of Arf1 could inhibit lipolysis in the digestive system of adult *Drosophila*, resulting in selective necrosis of normal and transformed stem cells. The author stated that the above phenomenon might provide new insights for the development of treatments for CSCs in human cancers (Singh et al., 2016). A study conducted by Wang et al. supported this notion, which showed that the knockdown of Arf1-pathway could kill CSCs effectively. The dying CSCs could be converted into therapeutic vaccines to stimulate a tumor-specific immune response, leading to sustaining benefits (Wang G. et al., 2020).

Interestingly, Pouyafar et al. found that the inhibition of lipolysis improved the EMT capacity of ovarian CSCs, while inhibiting glycolysis had an opposite result. The author pointed out that the potential EMT-promotion ability raised alertness of lipolysis inhibition application (Pouyafar et al., 2019).

#### Fatty Acid β-oxidation

Fatty acid oxidation (FAO) is a catabolic process that consumes long-chain fatty acids to provide ATP and NADPH, both of which promote cancer growth (Houten et al., 2016). In many cancer types, including colorectal, gastric, and breast cancer, accompanied by upregulated lipid synthesis and lipid uptake, FAO is also upregulated to maintain the balance of intracellular lipid (Carracedo et al., 2013; Camarda et al., 2016; Ma et al., 2018; Han et al., 2019). The elevated expressions of key rate-limiting FAO-related enzymes, such as fatty acyl-CoA synthetase, CPT1, carnitine palmitoyltransferase 2 (CPT2), are essential for the upregulation of FAO (Ma et al., 2018), which may herald a poor prognosis in acute myeloid leukemia and ovarian cancer (Shao et al., 2016; Shi et al., 2016).

Carnitine palmitoyltransferase 1 is the key rate-limiting enzyme of FAO, which transfers acyl-CoA from the cytoplasm into mitochondria and directly controls the rate of FAO and regulates cancer metabolic adaptation (Figure 1). Carnitine palmitoyltransferase 1 has three subtypes, CPT1A, CPT1B and CPT1C. Carnitine palmitoyltransferase 1A and CPT1B are widely distributed in the human body and show high similarities, while CPT1C is specifically expressed in the brain (Qu et al., 2016). The expressions of CPT1 and CPT2 are closely related to the ability of resistance to therapy, self-renewal, and metastasis in CSCs (Iwamoto et al., 2018; Yao et al., 2018; Han et al., 2019; Han et al., 2021).

More and more studies have shown that FAO plays an important role in mediating the resistance to cancer therapy, such as breast cancer and leukemia (Barger et al., 2013; Shinohara et al., 2016; Farge et al., 2017; Kitajima et al., 2017). The resistance to therapy is a major obstacle in cancer treatment, especially for patients with multi-organ metastases. It is reported that NANOG, a type of stem cell marker, can promote the stemness features of self-renewal and the resistance to therapy in CSCs by activating FAO. The downregulation of NANOG could make hepatocellular CSCs re-sensitive to sorafenib and inhibit the progression of hepatocellular carcinoma (Chen et al., 2016). In acute myeloid leukemia cells, increased expression of CD36 and FAO was found to be associated with the resistance to cytarabine (Farge et al., 2017). Wang et al. proved that blocking CPT1B by inhibiting JAK/STAT3 pathway could suppress the stemness of breast CSCs and re-sensitize breast CSCs to chemotherapy (Wang B. et al., 2018). Similarly, it has been found that the increased expressions of CPT1 and CPT2 are associated with the resistance to radiotherapy of breast cancer (Han et al., 2019). Besides, significant anti-angiogenic drug resistance has been found in adipose-associated tumors, including colorectal cancer and pancreatic ductal adenocarcinoma (PDAC). Blocking the function of CPT1A could re-sensitize tumor cells to anti-angiogenic drugs (Iwamoto et al., 2018). Furthermore, Nimmakayala et al. proved that FAO played an essential part in inducing oxidative phosphorylation in drug-resistant PDAC stem cells. Interestingly, in this study, the authors found that different subgroups of distant metastases of PDAC had different metabolic characteristics. Compared to lung metastasis cells, liver metastasis cells showed higher expression levels of FAO-related genes, such as CPT1A. It indicated that the innate metabolic features of different subpopulations of CSCs might determine the metastasis destinations of cancer (Nimmakayala et al., 2021).

Besides, the FAO pathway affects other functions of CSCs. The upregulation of FAO in CSCs improved the ability of metastasis by inducing EMT (Wang et al., 2019a), and enhanced the ability of self-renewal in breast CSCs (Wang T. et al., 2018). The expression level of fatty acyl-CoA synthetase VL3 (ACSVL3), an enzyme in the process of FAO, was significantly increased in glioblastoma CSCs and the knockdown of ACSVL3 inhibited the neurosphere-forming ability of glioblastoma. The author pointed out that ACSVL3 was a potential therapeutic target for glioblastoma because normal cells could survive and grow without ACSVL3 (Sun et al., 2014).

Accumulating studies have proved the relationship between resistance to therapy and FAO, which means the FAO inhibitor may become potential adjuvant therapy for therapy-resistant cancers and improve the prognosis of cancers (Table 1). In addition, the upregulation of FAO has been proved to support the stemness of CSCs and promote the progression of cancers. Based on these, drugs targeting to FAO have potential for cancer therapy.

#### Lipid Desaturation

In cancer cells, vigorous lipid synthesis and lipid uptake inevitably results in the accumulation of lipids, leading to lipotoxicity (Pinel et al., 2016). In addition to LDs, the lipid desaturation pathways can protect cancer cells from lipotoxicity and generate unsaturated lipids for the growth and proliferation of cancer cells (Mason et al., 2012; Peck et al., 2016). Unsaturated lipids are critical to the structure of cell membranes, which also improve the fluidity of cellular membranes (Peck and Schulze, 2016; Vanni et al., 2019).

Stearoyl-CoA desaturase is the key rate-limiting enzyme for lipid desaturation. It is a delta-9 fatty acid desaturase located in the endoplasmic reticulum membrane, which catalyzes saturated fatty acids (SFAs) to monounsaturated fatty acids (MUFAs) (Tracz-Gaszewska and Dobrzyn, 2019) (Figure 1). Monounsaturated fatty acids are essential materials for the biosynthesis of other unsaturated lipids, including polyunsaturated fatty acids (PUFAs), phospholipids (PL), and triglycerides, which are associated with cell growth, energy metabolism, and signal transduction. Stearoyl-CoA desaturase 1 is the main isoform of SCDs in humans and expresses in all tissues and organs widely (Zhang et al., 1999). It plays an important role in lipid desaturation and is the hotspot of current research. It has been observed that the expression of SCD1 is upregulated in hepatocellular, renal clear cell, lung, prostate and breast cancer, which is associated with poorer prognosis (Fritz et al., 2010; Holder et al., 2013; von Roemeling et al., 2013; Huang et al., 2015; Wohlhieter et al., 2020). Recent evidence also supported that SCD1 involved in many tumor-related pathways and played important roles in the self-renewal, metastasis, and resistance to therapy in glioblastoma, breast, lung and bladder CSCs (Noto et al., 2013; Colacino et al., 2016; Li J. et al., 2017; Mukherjee et al., 2017; Noto et al., 2017; Pisanu et al., 2018; Pinkham et al., 2019; Gao et al., 2020; Yu et al., 2021).

Based on these findings, SCD1 appears to be a significant participant in the development of malignancies and may be a promising target for anticancer therapy. Li et al. found the level of unsaturated lipids in ovarian CSCs was significantly elevated compared to non-stem cells of ovarian cancer. The reduction of lipid desaturation by inhibiting SCD1 could restrain the stemness of CSCs and cause the death of CSCs (Table 1). Experiments have also demonstrated that there is a positive feedback relationship between NF-κB signaling and SCD1, so we can suppress the expression of SCD1 by inhibiting the NF-κB signaling pathway to enhance cancer therapy (Li J. et al., 2017; Mukherjee et al., 2017). Besides, SCD1 inhibitors could be used as an adjuvant therapy to improve the sensitivity of CSCs to other antitumor drugs. In glioblastoma CSCs, by the inhibition of SCD1, the secondary accumulation of SFAs impaired DNA-repair mechanisms, and ultimately improved the efficacy of temozolomide (Pinkham et al., 2019). Similarly, in lung CSCs, the combined use of SCD1 inhibitors made therapy-resistant lung cancer re-sensitize to cisplatin (Pisanu et al., 2017).

Interestingly, Zhang et al. observed that SCD1 had a negative impact on the survival of leukemia CSCs. The inhibition of SCD1 accelerates the development of chronic myeloid leukemia (Zhang et al., 2012), which might indicate the potential differential role of SCD1 in solid tumor and hematological tumor. The metabolic plasticity of CSCs in solid tumors may explain this difference. Liver and lung carcinomas could produce unsaturated fatty acids by desaturating palmitate to sapienate, which was an unusual fatty acid, to resist the cellular damage caused by the inhibition of SCD1 (Vriens et al., 2019).

The pathway of lipid desaturation is essential to cell survival and is prevalently upregulated in many types of cancers. The key enzyme, SCD1, is upregulated in CSCs of many cancer types, which supported the growth of CSCs by providing MUFAs and protecting CSCs from lipotoxicity. Increasing evidence has proved that targeting SCD1 could inhibit the stemness of CSCs and suppress the progression of cancers. However, the existence of other lipid desaturation pathways suggests that only inhibiting SCD1 may not be enough to restrain the progression of some cancer lines.

#### Ferroptosis

**FIGURE 3:**
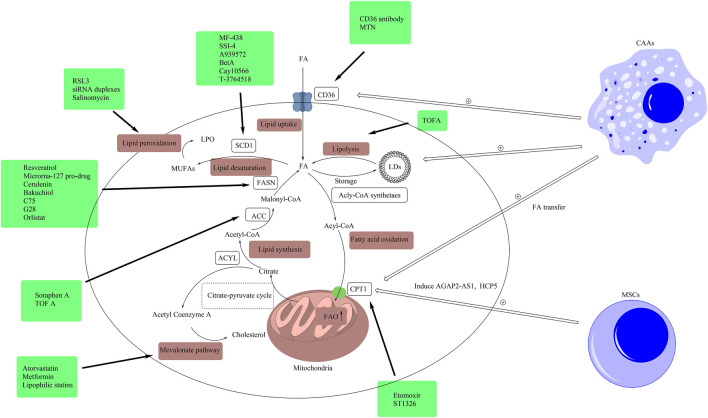
Drugs targeting lipid metabolism. In this figure, we presented related drugs, which were listed in Table 1, in the boxes and linked them to the targets of lipid metabolism including lipid uptake, lipolysis, fatty acid oxidation, lipid synthesis, lipid desaturation, the mevalonate pathway and lipid peroxidation. (ACLY, ATP citrate lyase; ACC, acetyl-CoA carboxylase; CAA, cancer-associated adipocytes; FA, fatty acid; FAO, fatty acid oxidation; FASN, fatty acid synthase; CD36, cluster of differentiation 36; LD, lipid droplet; CPT1, carnitine palmitoyltransferase-1; MUFA, monounsaturated fatty acid; MSC, mesenchymal stem cell; SCD1, stearoyl-CoA desaturase 1; TME, tumor microenvironment; LPO, lethal lipid peroxides).

Ferroptosis is an iron-induced, lipid-peroxide-driven form of programmed cell death (Dixon et al., 2012; Galluzzi et al., 2015; Li et al., 2019; Mou et al., 2019; Elgendy et al., 2020) (Table 1). The morphological features of dysmorphic smaller mitochondria with decreased and flat cristae, condensed mitochondrial membrane, and ruptured outer membrane help us distinguish ferroptosis from other forms of cell death (Dixon et al., 2012). Lethal lipid peroxide (LPO), which is cytotoxic, is the primary cause of ferroptosis. The production of LPO is mainly from two pathways, the pathways of auto-oxidation of lipids and enzymatic lipid peroxidation. The process of auto-oxidation of lipids, a free radical chain reaction, is usually initiated by ROS and leads to the accumulation of LPO (Ito et al., 2016; Hassannia et al., 2019; Ito et al., 2019; Yamada et al., 2020). In addition to the lipid peroxidation caused by ROS, lipid peroxides can be produced by enzymatic lipid peroxidation (Hassannia et al., 2019). Under the catalysis of acyl-CoA synthetase long-chain family member 4 (ACSL4), lysophosphatidylcholine acyltransferase 3 (LPCAT3), and 15-lipoxygenase (15LOX/ALOX15), arachidonoyl and adrenoyl phospholipids can be oxidized to produce LPO (Doll et al., 2017; Kagan et al., 2017) (Figure 3).

The elimination of LPO can protect cells from ferroptosis. The elimination of LPO relies on system X^−^_c_/GSH/GPX4 axis, NADPH/FSP1/coenzyme Q_10_ (CoQ_10_) axis, and other methods (Li and Li, 2020) (Figure 3). System X_C_^−^ is a glutamate/cystine anti-porter, which transports cystine into cells. Cystine is an important component of the synthesis of GSH. Glutathione peroxidase 4 (GPX4) is an enzyme that can reduce lipid hydroperoxides within biological membranes, which converts GSH to GSSH to reduce LPO and inhibit ferroptosis (Brigelius-Flohé and Maiorino, 2013; Li and Li, 2020). Besides, ferroptosis suppressor protein 1 (FSP1) catalyzes CoQ_10_ to ubiquinol by consuming NAD(P)H, which can reduce LPO (Doll et al., 2019; Li and Li, 2020). Glutathione peroxidase 4 and FSP1 have synergistic effects on anti-ferroptosis. It has been reported that the inhibition of GPX4 led to the upregulated expression of FSP1 to protect cells from ferroptosis (Bersuker et al., 2019).

In order to promote growth, cancer cells exhibit a higher iron requirement and more vigorous lipid metabolism than normal, non-cancer cells. These characteristics make cancer cells more vulnerable to ferroptosis (Hassannia et al., 2019; Yang Y. et al., 2020). Although the natural functions of ferroptosis remain unclear, ferroptosis shows great potential in cancer therapy, such as gastric, liver and pancreatic cancer (Zhang X. et al., 2019; Xu et al., 2019; Lee et al., 2020; Song et al., 2020; Zhang et al., 2020). For example, sorafenib as a system X_C_^−^ inhibitor can improve the prognosis of patients with advanced renal cell carcinoma and advanced hepatocellular carcinoma (Cheng et al., 2019). In addition, several studies have reported the induction of ferroptosis led to the suppression of the stemness of CSCs, including colorectal, breast, and glioblastoma CSCs (Buccarelli et al., 2018; Zhao et al., 2019; Xu et al., 2020). It has been reported that CSCs are less susceptible to death by classical apoptosis inducers (Elgendy et al., 2020). Therefore, ferroptosis provides us with a new way to treat cancer by inducing programmed cell death in cancer cells (Orlando et al., 2019). The increased susceptibility to ferroptosis is associated with the suppression of the stemness in CSCs. For example, Liu et al. found that the overexpression of the CSC marker CD44 was positively associated with the stability of SLC7A11, which is an important component of system X_C_^−^ (Liu T. et al., 2019). The regulations of key factors in the process of lipid peroxidation, such as inhibiting the expression of system X_C_^−^, GPX4, and FSP1, or enhancing the expression of ALOX15, or ACSL4, have been proved to be effective in the inhibition of CSCs stemness by inducing ferroptosis. Salinomycin exhibited a powerful ability to induce breast CSCs to ferroptosis by increasing iron accumulation and blocking the activity of GPX4 (Zhao et al., 2019). Additionally, it was reported that ionizing radiation induced the upregulated expression of ACSL4 and the accumulation of LPO, thereby, impairing and killing cancer cells. The absence of ACSL4 led to the resistance to radiotherapy in CSCs (Lei et al., 2020).

Ferroptosis is not only related to lipid peroxidation but also closely related to the entire lipid metabolism of CSCs. As mentioned above, the level of LDs and the expression of SCD1 are closely related to the stemness of CSCs, partly because of their protective effects against lipid peroxidation and ferroptosis. Lipid droplets prevent the contact and reaction of lipids with ROS (Stockwell et al., 2017). The involvement of SCD1 is essential for the conversion of SFAs to MUFAs, which is beneficial to the prevention of ferroptosis (Magtanong et al., 2019). Tesfay et al. observed that the inhibition of SCD1 significantly enhanced the anti-tumor effect of ferroptosis inducers in ovarian CSCs. The author pointed out that the combined therapy of SCD1 inhibitors and ferroptosis inducers had great potential in cancer treatment (Tesfay et al., 2019). Besides, the mevalonate pathway also played a protective role against ferroptosis by increasing the expression of GPX4 and CoQ_10_ (Stockwell et al., 2017). Based on these studies, the multiple combinations of the inducers of ferroptosis seem to have a better effect on cancer therapy (Figure 2).

Although the significant effects of ferroptosis in cancer therapy have been demonstrated in a series of experiments, making it a current research hotspot, several issues need to be considered before using ferroptosis inducers in clinical trials. First, the susceptibility to ferroptosis varies in different cancer cells. Compared to breast, colon, and lung cancers, renal cell carcinoma and diffuse large B cell lymphomas are more sensitive to ferroptosis (Mou et al., 2019), which indicates that not all cancer types are suitable for the treatment with ferroptosis inducers. Second, the direct or indirect inhibition or deactivation of GXP4 could lead to ferroptosis (Yang and Stockwell, 2016; Imai et al., 2017; Kajarabille and Latunde-Dada, 2019; Su et al., 2019). However, a study conducted by Bersuker et al. exhibited that the ferroptosis-resistant H460 lung cancer cells could grow normally with knockout of GPX4 in a preclinical tumor xenograft mouse model. The growth of tumors could be inhibited only by knocking out both GPX4 and FSP1 (Bersuker et al., 2019; Doll et al., 2019). Besides, Mannes et al. pointed out that even a minute level of GPX4 expression was sufficient for therapy-resistant cancer cells to survive, suggesting that only partial inhibition of the expressions of GPX4 and FSP1 might not be enough to kill CSCs in some cancers (Mannes et al., 2011). Third, ferroptosis is a complex process, and its function in normal cells is still unclear. The side effects of ferroptosis on normal cells must be considered. Therefore, studies focusing on the effects of ferroptosis in normal cells are needed. In conclusion, regulating ferroptosis in CSCs seems to have potential to improve cancer therapy, yet further research is still needed.

### The Signaling Pathways Associated With Lipid Metabolism in CSCs

In CSCs, the signaling pathways associated with lipid metabolism, such as Wnt, Notch, Hippo and Hedgehog signaling pathways, play important roles in the regulation of the stemness of CSCs. For example, the Wnt/β-catenin signaling pathway enhanced *de novo* lipogenesis by increasing the expression and activity of the key enzymes in *de novo* lipogenesis in breast cancer cells (Vergara et al., 2017). Wang et al. found that the knockdown of FZD7, a kind of Wnt receptor that drove the upregulation of GPX4, could make platinum-tolerant ovarian CSCs re-sensitive to platinum, restrain the stemness and induce CSCs to ferroptosis (Wang et al., 2021). Accumulating evidence has proved that several signaling pathways are closely related to SCD1. The inhibition of SCD1 led to the selective elimination of colon CSCs through suppressing Wnt and Notch signaling (Yu et al., 2021). The results showed that the crosstalk between SCD1 and Hippo pathway played an important role in maintaining the stemness features of the lung, gastric, and melanoma CSCs (Noto et al., 2017; Pisanu et al., 2018; Gao et al., 2020). Besides, the Notch signaling pathway mediated the homeostasis of liver cancer cells by controlling FAO by mediating the expression of the FAO-associated genes (Song et al., 2016). Hu et al. pointed out that cholesterol could activate Hedgehog signaling pathway by binding and/or modifying Smoothened receptor (a component of Hedgehog signaling) to support the stemness of CSCs (Hu and Song, 2019).

Signaling pathways are closely related with lipid metabolism and have synergistic effects on the mediation of the stemness in CSCs. Targeting signaling pathways can improve cancer therapy by directly inhibiting stemness or indirectly inhibiting stemness by regulating lipid metabolism in CSCs.

### The Transcription Factors and Non-coding RNAs Associated With Lipid Metabolism in CSCs

A series of transcription factors have been proved to play essential roles in regulating the CSCs stemness by modulating lipid metabolism. For example, sterol regulatory element-binding proteins (SREBPs) are key transcription factors that have a wide range of effects on lipid metabolism. They include three subtypes: SREBP-1a, SREBP-1c, and SREBP-2 (Cheng et al., 2018). The target genes of SREBPs are involved in the mediation of cholesterol uptake, biosynthesis, and fatty acid synthesis (Wen et al., 2018). Dysregulation of SREBPs occurs in various cancers. The knockdown of SREBP1 or SREBP2 restrained the expression of stemness-related genes and had an anti-proliferative effect on colon cancer cells (Wen et al., 2018). Besides, Lewis et al. found that the inhibition of SREBP blocked the expression of fatty acid-binding protein 7, which is a regulator of the functions of glioblastoma stem cells, and further impaired the survival of CSCs (Lewis et al., 2015). In addition to directly modulating SREBP, recent research has shown that it was feasible to restrain the stemness of CSCs by regulating the upstream pathways of SREBPs. The signaling pathways of peroxisome proliferator-activated receptor (PPARs) were positively associated with adipogenesis and lipid storage, and supported the mediation of stemness of CSCs, including breast, pancreatic, colorectal and hepatic CSCs (Wang et al., 2013; Ma et al., 2019; Kuramoto et al., 2021).

In addition to transcription factors, emerging evidence suggests that non-coding RNAs can mediate the stemness of CSCs by regulating lipid metabolism. For example, in triple-negative breast cancer, metformin could selectively eliminate CSCs by inducing the production of miRNA-193 family members. They induced apoptosis in CSCs by restraining the expression of FASN protein (Wahdan-Alaswad et al., 2014). Besides, another non-coding RNA, miRNA-328-3p, was found to suppress the stemness of breast cancer cells by modulating FAO. However, in ovarian CSCs, the inhibition of miRNA-328-3p restrained the stemness of CSCs, which was inconsistent with breast CSCs. The author pointed out that the function of miRNA-328-3p might differ in different tissues and cancer types (Zeng et al., 2021). Other non-coding RNAs, such as circular RNA, had been found to be involved in lipid metabolism, including FAO and fatty acid synthesis and influenced stemness properties of CSCs (Yu et al., 2019).

### Extracellular Signals Alterations IN CSCs

Apart from the intracellular lipid alterations in CSCs, the alterations of extracellular lipid signals in the TME also have significant effects on CSCs. The TME comprised of multiple stromal cells, including cancer-associated fibroblasts (CAFs), cancer-associated adipocytes (CAAs), mesenchymal stem cells (MSCs), immune cells and the extracellular matrix (ECM) as well (Quail and Joyce, 2013; Belli et al., 2018; Najafi et al., 2019; Terrén et al., 2019). Previous research has shown that there is a close relationship between cancer cells and components in the TME. These components regulate survival, growth, proliferation, invasion, metastasis, and resistance to therapy of cancer cells in numerous cancer types, such as hepatic, pancreatic, lung cancer and lymphomas (Wu et al., 2019b; Wang S. et al., 2020; Ling et al., 2021; Menter et al., 2021). Furthermore, many studies targeting stromal cells, immune cells, and ECM have proved that cancer can be inhibited and CSCs stemness can be impaired by regulating the TME, including targeting lipid signals in components of TME, especially in CAFs, CAAs, and MSCs.

### The Lipid Metabolism Rewiring on the Cell Communications Within the TME

#### Cancer-associated Fibroblasts

Cancer-associated fibroblasts are the most plentiful cells among stromal cells in the TME and are related to cancer progression, such as gastric and liver cancer (Kubo et al., 2016; Chen and Song, 2019; Kobayashi et al., 2019). Nowadays, accumulating studies have shown that CAFs supported the stemness of CSCs. For example, Zhang et al. found that CAFs secreted miR-522 to inhibit ALOX-15 in gastric cancer cells and suppressed ferroptosis. Furthermore, cisplatin and paclitaxel promoted the secretion of miR-522 in CAFs, which suppressed ferroptosis and improved chemoresistance (Zhang et al., 2020) (Figure 1 and Table 1).

Cancer-associated fibroblasts promote the survival of CSCs by suppressing ferroptosis. However, studies targeting lipid metabolism in CAFs are still lacking, which may offer insights into potential therapeutic strategies in cancer treatment.

#### Cancer-associated Adipocytes

Cancer-associated adipocytes are a subpopulation of adipocytes that support the progression of cancer. They can be found in tumor tissues, or in peritumoral regions of tumors, and even in distal tissues. Based on that, CAAs are divided into intratumoral adipocytes and peritumoral adipocytes (Cao, 2019).

Increasing evidence has proved that CAAs played important roles in the progression of cancers, such as breast and pancreatic cancer (Wu et al., 2019a; Cai et al., 2019; Takehara et al., 2020; Zhao et al., 2020). They promoted the self-renewal of CSCs and the progression of cancer. It was reported that CAAs promoted the self-renewal and proliferation of prostate CSCs by secreting cathepsin B. In breast cancer, Wang et al. found that co-cultured adipocytes acted as the reservoir of lipids and transferred FAs to breast CSCs to support the growth of breast CSCs. Furthermore, fatty acids transferred from adipocytes to breast CSCs induced the upregulated expression of CPT1B to improve stemness (Wang T. et al., 2018) (Figure 1).

Besides, CAAs are associated with the resistance of CSCs to therapy. Chi et al. found that the co-culture of adipocytes and melanoma cells enhanced the chemoresistance to cisplatin and docetaxel (Chi et al., 2014). In breast and pancreatic cancer, researchers have observed a similar phenomenon in chemoresistance to gemcitabine (De Angel et al., 2013; Okumura et al., 2017). The ability of CAAs to enhance resistance to therapy may be due to its secreted factors. Leptin was reported to inhibit the sensitivity to hormonal therapy in breast cancer (Delort et al., 2019; Kitson et al., 2019).

In addition, CSCs could transform normal adipocytes to support their growth, which was observed in a recent study based on a mouse model of blast crisis chronic myeloid leukemia. A subpopulation of leukemic CSCs had an ability to induce the lipolysis of gonadal adipose tissue cells and provided free fatty acid to leukemic CSCs for metabolism, especially the subpopulation of CD36^+^ leukemic CSCs. The author speculated that the leukemic CSCs transformed gonadal adipose tissue into a niche to support their survival and evade chemotherapy (Ye et al., 2016).

Moreover, epidemiological research has shown that the obese people are more likely to suffer from cancers, including colorectal, breast, prostate, gastric, thyroid, pancreatic, and hepatic cancers (Allott et al., 2013; Bardou et al., 2013; De Pergola and Silvestris, 2013; Ilic and Ilic, 2016; Engin, 2017; Avgerinos et al., 2019; Saitta et al., 2019; Matrone et al., 2020). Sametime, obesity is associated with poorer prognosis in breast and colorectal cancer (Bardou et al., 2013; Jiralerspong and Goodwin, 2016). Besides, free fatty acids excreted by host cells also promoted the growth of tumor cells (Martinez-Outschoorn et al., 2011). Obese adipose tissues are more beneficial to cancer cells to grow compared with their lean counterparts by providing more lipids and limiting drug perfusion (Cao, 2019). It has been reported that obese adipocytes upregulated the expression of CD36 in ovarian CSCs to support the stemness of CSCs (Ladanyi et al., 2018).

In short, CAAs act as a lipids storage for CSCs to support their growth and secrete cell factors to support the stemness of CSCs. Obesity is a risk factor for cancer, which is related to the higher incidence and poorer prognosis of cancer, which possibly results from higher contents of adipocytes in obese patients.

#### Mesenchymal Stem Cells

Mesenchymal stem cells are a type of multipotent stem cells and play important roles in regeneration and wound healing (Li et al., 2019). However, in the TME, MSCs are transformed to support the growth of cancer cells and promote cancer progression (Ono et al., 2014; Zhang X. et al., 2018; Chen et al., 2019; Liu Y. et al., 2019). Studies have shown that MSCs could regulate the stemness of CSCs by reconnecting lipid metabolism. For example, researchers reported MSCs could induce the expressions of AGAP2-AS1 and HCP5, the long non-coding RNAs, thereby promoting stemness and resistance to therapy in gastric and breast cancer by elevating FAO (He et al., 2019; Wu H. et al., 2020; Han et al., 2021) (Figure 1). Besides, MSCs could influence CSCs by mediating immune cells in TME. The MSCs of acute myeloid leukemia had higher expression of cyclooxygenase, which was the key enzyme in the production of prostaglandin D2 (PGD2). Prostaglandin D2 and its receptor prostaglandin D2 receptor 2 (PTGDR2) promoted the proliferation of malignant hematopoietic stem and progenitor cells by promoting the expansion of immune cells including type 2 innate lymphoid cells and CD4^+^CD25+IL5Rα+ T regulatory cells and promoting the production of cytokines including Interleukin-5, which could be inhibited by the blockage of PTGDR2 (Wu L. et al., 2020). In gastric CSCs, the expressions of PGD2 synthase and PTGDR2 were downregulated, leading to the upregulated expression of CSC markers and enhanced ability of self-renewal (Zhang B. et al., 2018).

The cooperation with CSCs and MSCs has improved the stemness of CSCs and drives cancer progression. Some cell factors, such as AGAP2-AS1, HCP5, serve as potential targets to inhibit the progression of cancer and improve the chemotherapy efficacy in cancer.

### Systematic Extracellular Lipids

In the TME, the exogenous lipids support the survival and growth of cancer cells. Accumulating evidence indicated that obesity was associated with higher cancer risk and poorer prognosis (Park et al., 2014; Hopkins et al., 2016; Avgerinos et al., 2019). Obesity-associated protein, an RNA N6-methyladenosine demethylase, had been identified to promote obesity and play oncogenic roles in cancer. Su et al. found that the inhibition of obesity-associated protein could restrain the ability of self-renewal in leukemia CSCs and reprogram immune response of leukemia CSCs by suppressing expression of immune checkpoint genes (Su et al., 2020). Tesfay et al. found that providing exogenous palmitoleic acid or oleate to ovarian cancer cells could protect them from ferroptosis (Tesfay et al., 2019). Besides, exogenous lipids could positively modulate Notch signaling, which plays a critical role in CSCs proliferation (Li et al., 2020).

The high lipid diet has been proved to promote tumorigenesis and cancer progression, including melanoma and breast cancer (Pascual et al., 2017; Wang B. et al., 2018). The high cholesterol diet was found to support the proliferation of intestinal stem cells, which proved to be the initiating cells of intestinal tumors, and promote the tumorigenesis (Wang B. et al., 2018). Beyaz et al. found that high-fat diet enhanced the stemness and tumor-initiating potential of intestinal CSCs by inducing the expression of transcription factor PPAR δ (Beyaz et al., 2016), which was found to mediate the effect of a high-fat diet in promoting liver metastasis by inducing the expansion of colonic CSCs (Wang et al., 2019b).

Systematic exogenous lipids also play important roles in the mediation of stemness in CSCs. The regulations of exogenous lipids are beneficial to cancer therapy.

## Conclusion and Perspectives

Cancer stem cells are a subpopulation of cancer cells that play significant roles in promoting cancer progression and are the principal causes of resistance to therapy, relapse, and metastasis (Batlle and Clevers, 2017). Lipid signals are widely altered in the CSCs of numerous cancer types, by which CSCs gain survival advantages and promote cancer progression. In this review, we described the influence of intracellular lipid signals, including lipid droplets contents, lipid uptake, lipolysis, fatty acid oxidation, lipid desaturation, lipid peroxidation of CSCs, and the influence of lipid signals on components in the TME on CSCs. Generally, the upregulation of intracellular lipid metabolism, except for lipid peroxidation, plays important roles in maintaining the stemness of CSCs and promoting the growth and progression of cancer and is correlated with poor prognosis. The elements in TME, such as CAFs, CAAs, MSCs, have supportive effects on the maintenance of CSCs stemness by reconnecting lipid signals. More and more studies have proved that targeting lipid signals is a potential treatment for cancer therapy. The regulations of key enzymes in lipid metabolism or ferroptosis could exhibit anti-tumor effects in CSCs and cancer cells, including restraining the ability of self-renewal, growth, metastasis of CSCs, inducing ferroptosis, and reversing the resistance to therapy of CSCs, offering opportunities to develop novel drugs for cancer treatment. Inhibition of the extracellular lipid signals impairs the supportive effects of TME on CSCs.

Although it sounds promising, we must be careful because cholesterol and fatty acids are the fundamental energy sources for almost all the cells and are the precursors for a wide variety of molecules that play remarkable biological roles. It is necessary to identify specific targets to inhibit lipid signals only in CSCs without affecting normal cells that generally use lipids to produce energy. For example, muscle stem cells are dependent on mitochondrial fatty acid oxidation and oxidative phosphorylation and intestinal stem cells highly rely on fatty acid oxidation in their maintenance (Mihaylova et al., 2018). The molecular basis of the differences regarding lipid metabolism in CSCs and normal cells remains poorly understood. So far, most drugs targeting lipid metabolism are still in preclinical research, and few studies have mentioned the side effects of the drugs targeting lipid metabolism on normal cells. Therefore, more efforts are needed to fulfill the transformation of this potential therapeutic approach into the clinic. The combination of the inhibitors of lipid metabolism with a targeted drug delivery system may serve as an alternative.

Some signaling pathways have been reported to regulate both lipid metabolism and stemness, for example, AMPK pathway. Active AMPK promotes the oxidation of fatty acids and inhibits the synthesis of fatty acids and cholesterol, which involves largely in acetyl-CoA. The AMPK pathway also phosphorylates and inhibits HMGCR, which requires acetyl-CoA during the reduction reaction (Koo and Guan, 2018). This offers insights into reducing tumor energy supply by targeting AMPK signaling. However, AMPK can directly phosphorylate YAP and inhibit its transcriptional activity. The AMPK pathway is tightly involved in cancer drug resistance by regulating ABCG2 expression (Wang et al., 2016). The inhibitors of AMPK have been reported to promote epithelial-to-mesenchymal transition in breast and prostate cancer (Yuan et al., 2020). Therefore, depending on conditions, whether AMPK inhibition therapy suppresses or promotes cancer remains ambiguous. When developing pathway-specific activators/inhibitors, although they sound attractive and promising, yet cautions must be taken to evaluate its effects systematically in the long run.

The heterogeneity of CSCs must be taken into consideration. Lipid metabolism exerts varying effects on different cancer types or even different subtypes of certain cancer. The inhibition of lipolysis increased EMT-associated genes in ovarian stem cells. Unlike most of the cancer types, SCD1 plays a tumor-suppressive role in leukemia stem cells with no effect on the function of normal hematopoietic stem cells (Zhang et al., 2012; Pouyafar et al., 2019). These data suggest that we cannot simply extrapolate the conclusions applicable from one cancer to another cancer.

At present, research on extracellular lipid signals is too scarce to further prove the feasibility of this therapeutic method for cancer therapy. Further consideration and research are needed to prove the effectiveness and safety of anti-tumor drugs based on lipid signals. However, in summary, targeting lipid signals gives us a new way to cure cancers.

## Glossary

AA: Arachidonoyl
ACACA: Acetyl-CoA carboxylase A
ACC: Acetyl-CoA carboxylase
ACLY: ATP-citrate lyase
ACSL4: Acyl-CoA synthetase long-chain family member 4
ACSVL3: Acyl-CoA synthetase VL3
AdA: Adrenoyl
AML: Acute myelocytic leukemia
Arf1: ADP-ribosylation factor 1
ATP: Adenosine triphosphate
CAA: Cancer-associated adipocyte
CAF: Cancer-associated fibroblast
CE: Cholesterol ester
CoQ_10_: Coenzyme Q_10_
CPT: Carnitine palmitoyltransferase
CSC: Cancer stem cell
ECM: Extracellular matrix
EMT: Epithelial-mesenchymal transition
FA: Fatty acid
FAO: Fatty acid oxidation
FASN: Fatty acid synthase
FSP1: Ferroptosis suppressor protein 1
GPX4: Glutathione peroxidase 4
GSH: Glutathione
HMGCR: Hydroxy-3-methylglutaryl CoA reductase
HMGCS1: 3-hydroxy-3-methylglutaryl-CoA synthase 1
LD: Lipid droplet
LDL: Low-density lipoprotein
LPCAT3: Lysophosphatidylcholine acyltransferase 3
LPO: Lethal lipid peroxide
MSC: Mesenchymal stem cell
MTN: 2-methylthio-1,4-naphthoquinone
MUFA: Monounsaturated fatty acid
NADPH: Nicotinamide adenine dinucleotide phosphate
PDAC: Pancreatic ductal adenocarcinoma
PGD2: Prostaglandin D2
PL: Phospholipid
PPAR: Peroxisome proliferator-activated receptor
PTGDR2: Prostaglandin D2 receptor 2
PUFA: Polyunsaturated fatty acid
ROS: Reactive oxygen species
SCD: Stearoyl-CoA desaturase
SFA: Saturated fatty acid
SQLE: Squalene epoxidase
SREBP: Sterol regulatory element-binding protein
TG: Triglyceride
TIC: Tumor initiating cell
TME: Tumor microenvironment

## References

[B1] Al-Bahlani S. Al-Lawati H. Al-Adawi M. Al-Abri N. Al-Dhahli B. Al-Adawi K. (2017). Fatty Acid Synthase Regulates the Chemosensitivity of Breast Cancer Cells to Cisplatin-Induced Apoptosis. Apoptosis 22 (6), 865–876. 10.1007/s10495-017-1366-2 28386750

[B2] Ali A. Levantini E. Teo J. T. Goggi J. Clohessy J. G. Wu C. S. (2018). Fatty Acid Synthase Mediates EGFR Palmitoylation in EGFR Mutated Non-small Cell Lung Cancer. EMBO Mol. Med. 10 (3), e8313. 10.15252/emmm.201708313 29449326 PMC5840543

[B3] Allott E. H. Masko E. M. Freedland S. J. (2013). Obesity and Prostate Cancer: Weighing the Evidence. Eur. Urol. 63 (5), 800–809. 10.1016/j.eururo.2012.11.013 23219374 PMC3597763

[B4] Ameer F. Scandiuzzi L. Hasnain S. Kalbacher H. Zaidi N. (2014). De Novo Lipogenesis in Health and Disease. Metabolism 63 (7), 895–902. 10.1016/j.metabol.2014.04.003 24814684

[B5] Arneth B. (2019). Tumor Microenvironment. Medicina (Kaunas) 56 (1), 15. 10.3390/medicina56010015 PMC7023392 31906017

[B6] Avgerinos K. I. Spyrou N. Mantzoros C. S. Dalamaga M. (2019). Obesity and Cancer Risk: Emerging Biological Mechanisms and Perspectives. Metabolism 92, 121–135. 10.1016/j.metabol.2018.11.001 30445141

[B7] Bader J. E. Voss K. Rathmell J. C. (2020). Targeting Metabolism to Improve the Tumor Microenvironment for Cancer Immunotherapy. Mol. Cel 78 (6), 1019–1033. 10.1016/j.molcel.2020.05.034 PMC7339967 32559423

[B8] Bailey A. P. Koster G. Guillermier C. Hirst E. M. MacRae J. I. Lechene C. P. (2015). Antioxidant Role for Lipid Droplets in a Stem Cell Niche of Drosophila. Cell 163 (2), 340–353. 10.1016/j.cell.2015.09.020 26451484 PMC4601084

[B9] Bardou M. Barkun A. N. Martel M. (2013). Obesity and Colorectal Cancer. Gut 62 (6), 933–947. 10.1136/gutjnl-2013-304701 23481261

[B10] Barger J. F. Gallo C. A. Tandon P. Liu H. Sullivan A. Grimes H. L. (2013). S6K1 Determines the Metabolic Requirements for BCR-ABL Survival. Oncogene 32 (4), 453–461. 10.1038/onc.2012.70 22391570 PMC3371300

[B11] Barreno L. Cáceres S. Alonso-Diez Á. Vicente-Montaña A. García M. L. Clemente M. (2019). Vasculogenic Mimicry-Associated Ultrastructural Findings in Human and Canine Inflammatory Breast Cancer Cell Lines. BMC Cancer 19 (1), 750. 10.1186/s12885-019-5955-z 31362745 PMC6668131

[B12] Batlle E. Clevers H. (2017). Cancer Stem Cells Revisited. Nat. Med. 23 (10), 1124–1134. 10.1038/nm.4409 28985214

[B13] Belli C. Trapani D. Viale G. D'Amico P. Duso B. A. Della Vigna P. (2018). Targeting the Microenvironment in Solid Tumors. Cancer Treat. Rev. 65, 22–32. 10.1016/j.ctrv.2018.02.004 29502037

[B14] Bergroth E. Aakula M. Korppi M. Remes S. Kivistö J. E. Piedra P. A. (2016). Post-bronchiolitis Use of Asthma Medication: A Prospective 1-year Follow-Up Study. Pediatr. Infect. Dis. J. 35 (4), 363–368. 10.1097/inf.0000000000001017 26658529

[B15] Bersuker K. Hendricks J. M. Li Z. Magtanong L. Ford B. Tang P. H. (2019). The CoQ Oxidoreductase FSP1 Acts Parallel to GPX4 to Inhibit Ferroptosis. Nature 575 (7784), 688–692. 10.1038/s41586-019-1705-2 31634900 PMC6883167

[B16] Beyaz S. Mana M. D. Roper J. Kedrin D. Saadatpour A. Hong S. J. (2016). High-fat Diet Enhances Stemness and Tumorigenicity of Intestinal Progenitors. Nature 531 (7592), 53–58. 10.1038/nature17173 26935695 PMC4846772

[B17] Brandi J. Dando I. Pozza E. D. Biondani G. Jenkins R. Elliott V. (2017). Proteomic Analysis of Pancreatic Cancer Stem Cells: Functional Role of Fatty Acid Synthesis and Mevalonate Pathways. J. Proteomics 150, 310–322. 10.1016/j.jprot.2016.10.002 27746256

[B18] Brigelius-Flohé R. Maiorino M. (2013). Glutathione Peroxidases. Biochim. Biophys. Acta (Bba) - Gen. Subjects 1830 (5), 3289–3303. 10.1016/j.bbagen.2012.11.020 23201771

[B19] Buccarelli M. Marconi M. Pacioni S. De Pascalis I. D'Alessandris Q. G. Martini M. (2018). Inhibition of Autophagy Increases Susceptibility of Glioblastoma Stem Cells to Temozolomide by Igniting Ferroptosis. Cell Death Dis 9 (8), 841. 10.1038/s41419-018-0864-7 30082680 PMC6079099

[B20] Cai Z. Liang Y. Xing C. Wang H. Hu P. Li J. (2019). Cancer-associated A-dipocytes E-xhibit D-istinct P-henotypes and F-acilitate T-umor P-rogression in P-ancreatic C-ancer. Oncol. Rep. 42 (6), 2537–2549. 10.3892/or.2019.7365 31638193 PMC6826327

[B21] Camarda R. Zhou A. Y. Kohnz R. A. Balakrishnan S. Mahieu C. Anderton B. (2016). Inhibition of Fatty Acid Oxidation as a Therapy for MYC-Overexpressing Triple-Negative Breast Cancer. Nat. Med. 22 (4), 427–432. 10.1038/nm.4055 26950360 PMC4892846

[B22] Cancer Genome Atlas Research N. (2015). The Molecular Taxonomy of Primary Prostate Cancer. Cell 163 (4), 1011–1025. 10.1016/j.cell.2015.10.025 26544944 PMC4695400

[B23] Cao Y. (2019). Adipocyte and Lipid Metabolism in Cancer Drug Resistance. J. Clin. Invest. 129 (8), 3006–3017. 10.1172/JCI127201 31264969 PMC6668696

[B24] Carracedo A. Cantley L. C. Pandolfi P. P. (2013). Cancer Metabolism: Fatty Acid Oxidation in the Limelight. Nat. Rev. Cancer 13 (4), 227–232. 10.1038/nrc3483 23446547 PMC3766957

[B25] Chen C. L. Uthaya Kumar D. B. Punj V. Xu J. Sher L. Tahara S. M. (2016). NANOG Metabolically Reprograms Tumor-Initiating Stem-like Cells Through Tumorigenic Changes in Oxidative Phosphorylation and Fatty Acid Metabolism. Cell Metab 23 (1), 206–219. 10.1016/j.cmet.2015.12.004 26724859 PMC4715587

[B26] Chen X. Song E. (2019). Turning Foes to Friends: Targeting Cancer-Associated Fibroblasts. Nat. Rev. Drug Discov. 18 (2), 99–115. 10.1038/s41573-018-0004-1 30470818

[B27] Chen Y. C. Gonzalez M. E. Burman B. Zhao X. Anwar T. Tran M. (2019). Mesenchymal Stem/Stromal Cell Engulfment Reveals Metastatic Advantage in Breast Cancer. Cell Rep 27 (13), 3916–3926.e5. 10.1016/j.celrep.2019.05.084 31242423 PMC6657699

[B28] Cheng C. Geng F. Cheng X. Guo D. (2018). Lipid Metabolism Reprogramming and its Potential Targets in Cancer. Cancer Commun. (Lond) 38 (1), 27. 10.1186/s40880-018-0301-4 29784041 PMC5993136

[B29] Cheng Y. Tang X. Y. Li Y. X. Zhao D. D. Cao Q. H. Wu H. X. (2019). Depression-Induced Neuropeptide Y Secretion Promotes Prostate Cancer Growth by Recruiting Myeloid Cells. Clin. Cancer Res. 25 (8), 2621–2632. 10.1158/1078-0432.CCR-18-2912 30504424

[B30] Chi M. Chen J. Ye Y. Tseng H. Y. Lai F. Tay K. H. (2014). Adipocytes Contribute to Resistance of Human Melanoma Cells to Chemotherapy and Targeted Therapy. Curr. Med. Chem. 21 (10), 1255–1267. 10.2174/0929867321666131129114742 24304284

[B31] Cilibrasi C. Riva G. Romano G. Cadamuro M. Bazzoni R. Butta V. (2017). Resveratrol Impairs Glioma Stem Cells Proliferation and Motility by Modulating the Wnt Signaling Pathway. PLoS One 12 (1), e0169854. 10.1371/journal.pone.0169854 28081224 PMC5231344

[B32] Colacino J. A. McDermott S. P. Sartor M. A. Wicha M. S. Rozek L. S. (2016). Transcriptomic Profiling of Curcumin-Treated Human Breast Stem Cells Identifies a Role for Stearoyl-Coa Desaturase in Breast Cancer Prevention. Breast Cancer Res. Treat. 158 (1), 29–41. 10.1007/s10549-016-3854-4 27306423 PMC5831404

[B33] Cruz A. L. S. Barreto E. A. Fazolini N. P. B. Viola J. P. B. Bozza P. T. (2020). Lipid Droplets: Platforms with Multiple Functions in Cancer Hallmarks. Cel Death Dis 11 (2), 105. 10.1038/s41419-020-2297-3 PMC7005265 32029741

[B34] Daniel Y. Lelou E. Aninat C. Corlu A. Cabillic F. (2021). Interplay Between Metabolism Reprogramming and Epithelial-To-Mesenchymal Transition in Cancer Stem Cells. Cancers (Basel) 13 (8), 1973. 10.3390/cancers13081973 33923958 PMC8072988

[B35] Dattilo R. Mottini C. Camera E. Lamolinara A. Auslander N. Doglioni G. (2020). Pyrvinium Pamoate Induces Death of Triple-Negative Breast Cancer Stem-like Cells and Reduces Metastases Through Effects on Lipid Anabolism. Cancer Res. 80 (19), 4087–4102. 10.1158/0008-5472.Can-19-1184 32718996 PMC8808379

[B36] De Angel R. E. Blando J. M. Hogan M. G. Sandoval M. A. Lansakara-P D. S. Dunlap S. M. (2013). Stearoyl Gemcitabine Nanoparticles Overcome Obesity-Induced Cancer Cell Resistance to Gemcitabine in a Mouse Postmenopausal Breast Cancer Model. Cancer Biol. Ther. 14 (4), 357–364. 10.4161/cbt.23623 23358472 PMC3667876

[B37] De Pergola G. Silvestris F. (2013). Obesity as a Major Risk Factor for Cancer. J. Obes. 2013, 291546. 10.1155/2013/291546 24073332 PMC3773450

[B38] DeBose-Boyd R. A. (2018). Significance and Regulation of Lipid Metabolism. Semin. Cel Dev Biol 81, 97. 10.1016/j.semcdb.2017.12.003 29246858

[B39] Delort L. Bougaret L. Cholet J. Vermerie M. Billard H. Decombat C. (2019). Hormonal Therapy Resistance and Breast Cancer: Involvement of Adipocytes and Leptin. Nutrients 11 (12), 2839. 10.3390/nu11122839 PMC6950701 31756890

[B40] Dixon S. J. Lemberg K. M. Lamprecht M. R. Skouta R. Zaitsev E. M. Gleason C. E. (2012). Ferroptosis: An Iron-dependent Form of Nonapoptotic Cell Death. Cell 149 (5), 1060–1072. 10.1016/j.cell.2012.03.042 22632970 PMC3367386

[B41] Doll S. Freitas F. P. Shah R. Aldrovandi M. da Silva M. C. Ingold I. (2019). FSP1 Is a Glutathione-independent Ferroptosis Suppressor. Nature 575 (7784), 693–698. 10.1038/s41586-019-1707-0 31634899

[B42] Doll S. Proneth B. Tyurina Y. Y. Panzilius E. Kobayashi S. Ingold I. (2017). ACSL4 Dictates Ferroptosis Sensitivity by Shaping Cellular Lipid Composition. Nat. Chem. Biol. 13 (1), 91–98. 10.1038/nchembio.2239 27842070 PMC5610546

[B43] Elgendy S. M. Alyammahi S. K. Alhamad D. W. Abdin S. M. Omar H. A. (2020). Ferroptosis: An Emerging Approach for Targeting Cancer Stem Cells and Drug Resistance. Crit. Rev. Oncol. Hematol. 155, 103095. 10.1016/j.critrevonc.2020.103095 32927333

[B44] Engin A. (2017). Obesity-associated Breast Cancer: Analysis of Risk Factors. Adv. Exp. Med. Biol. 960, 571–606. 10.1007/978-3-319-48382-5_25 28585217

[B45] Farge T. Saland E. de Toni F. Aroua N. Hosseini M. Perry R. (2017). Chemotherapy-Resistant Human Acute Myeloid Leukemia Cells Are Not Enriched for Leukemic Stem Cells But Require Oxidative Metabolism. Cancer Discov. 7 (7), 716–735. 10.1158/2159-8290.CD-16-0441 28416471 PMC5501738

[B46] Folmes C. D. Terzic A. (2016). Energy Metabolism in the Acquisition and Maintenance of Stemness. Semin. Cel Dev Biol 52, 68–75. 10.1016/j.semcdb.2016.02.010 PMC4905551 26868758

[B47] Fritz V. Benfodda Z. Rodier G. Henriquet C. Iborra F. Avancès C. (2010). Abrogation of De Novo Lipogenesis by Stearoyl-CoA Desaturase 1 Inhibition Interferes with Oncogenic Signaling and Blocks Prostate Cancer Progression in Mice. Mol. Cancer Ther. 9 (6), 1740–1754. 10.1158/1535-7163.Mct-09-1064 20530718 PMC3315476

[B48] Gao M. Deng J. Liu F. Fan A. Wang Y. Wu H. (2019). Triggered Ferroptotic Polymer Micelles for Reversing Multidrug Resistance to Chemotherapy. Biomaterials 223, 119486. 10.1016/j.biomaterials.2019.119486 31520887

[B49] Gao Y. Li J. Xi H. Cui J. Zhang K. Zhang J. (2020). Stearoyl-CoA-desaturase-1 Regulates Gastric Cancer Stem-like Properties and Promotes Tumour Metastasis via Hippo/YAP Pathway. Br. J. Cancer 122 (12), 1837–1847. 10.1038/s41416-020-0827-5 32350414 PMC7283337

[B50] Garcia-Bermudez J. Baudrier L. Bayraktar E. C. Shen Y. La K. Guarecuco R. (2019). Squalene Accumulation in Cholesterol Auxotrophic Lymphomas Prevents Oxidative Cell Death. Nature 567 (7746), 118–122. 10.1038/s41586-019-0945-5 30760928 PMC6405297

[B51] Ghoneum A. Gonzalez D. Abdulfattah A. Y. Said N. (2020). Metabolic Plasticity in Ovarian Cancer Stem Cells. Cancers (Basel) 12 (5). 10.3390/cancers12051267 PMC7281273 32429566

[B52] Giampietri C. Petrungaro S. Cordella M. Tabolacci C. Tomaipitinca L. Facchiano A. (2017). Lipid Storage and Autophagy in Melanoma Cancer Cells. Int. J. Mol. Sci. 18 (6), 1271. 10.3390/ijms18061271 PMC5486093 28617309

[B53] Ginestier C. Monville F. Wicinski J. Cabaud O. Cervera N. Josselin E. (2012). Mevalonate Metabolism Regulates Basal Breast Cancer Stem Cells and Is a Potential Therapeutic Target. Stem Cells 30 (7), 1327–1337. 10.1002/stem.1122 22605458

[B54] Giró-Perafita A. Rabionet M. Planas M. Feliu L. Ciurana J. Ruiz-Martínez S. (2019). EGCG-derivative G28 Shows High Efficacy Inhibiting the Mammosphere-Forming Capacity of Sensitive and Resistant TNBC Models. Molecules 24 (6), 1027. 10.3390/molecules24061027 PMC6471537 30875891

[B55] Gopal K. Grossi E. Paoletti P. Usardi M. (1963). Lipid Composition of Human Intracranial Tumors: A Biochemical Study. Acta Neurochir (Wien) 11, 333–347. 10.1007/bf01402012 14064798

[B56] Hale J. S. Otvos B. Sinyuk M. Alvarado A. G. Hitomi M. Stoltz K. (2014). Cancer Stem Cell-specific Scavenger Receptor CD36 Drives Glioblastoma Progression. Stem Cells 32 (7), 1746–1758. 10.1002/stem.1716 24737733 PMC4063873

[B57] Han J. Qu H. Han M. Ding Y. Xie M. Hu J. (2021). MSC-induced lncRNA AGAP2-AS1 Promotes Stemness and Trastuzumab Resistance Through Regulating CPT1 Expression and Fatty Acid Oxidation in Breast Cancer. Oncogene 40 (4), 833–847. 10.1038/s41388-020-01574-8 33273726

[B58] Han S. Wei R. Zhang X. Jiang N. Fan M. Huang J. H. (2019). CPT1A/2-Mediated FAO Enhancement-A Metabolic Target in Radioresistant Breast Cancer. Front. Oncol. 9, 1201. 10.3389/fonc.2019.01201 31803610 PMC6873486

[B59] Hanai J. I. Doro N. Seth P. Sukhatme V. P. (2013). ATP Citrate Lyase Knockdown Impacts Cancer Stem Cells In Vitro. Cel Death Dis 4 (6), e696. 10.1038/cddis.2013.215 PMC3702307 23807225

[B60] Hassannia B. Vandenabeele P. Vanden Berghe T. (2019). Targeting Ferroptosis to Iron Out Cancer. Cancer Cell 35 (6), 830–849. 10.1016/j.ccell.2019.04.002 31105042

[B61] He W. Liang B. Wang C. Li S. Zhao Y. Huang Q. (2019). MSC-regulated lncRNA MACC1-AS1 Promotes Stemness and Chemoresistance Through Fatty Acid Oxidation in Gastric Cancer. Oncogene 38 (23), 4637–4654. 10.1038/s41388-019-0747-0 30742067 PMC6756048

[B62] Hershey B. J. Vazzana R. Joppi D. L. Havas K. M. (2019). Lipid Droplets Define a Sub-population of Breast Cancer Stem Cells. J. Clin. Med. 9 (1), 87. 10.3390/jcm9010087 PMC7019257 31905780

[B63] Holder A. M. Gonzalez-Angulo A. M. Chen H. Akcakanat A. Do K. A. Fraser Symmans W. (2013). High Stearoyl-CoA Desaturase 1 Expression Is Associated with Shorter Survival in Breast Cancer Patients. Breast Cancer Res. Treat. 137 (1), 319–327. 10.1007/s10549-012-2354-4 23208590 PMC3556743

[B64] Hopkins B. D. Goncalves M. D. Cantley L. C. (2016). Obesity and Cancer Mechanisms: Cancer Metabolism. J. Clin. Oncol. 34 (35), 4277–4283. 10.1200/jco.2016.67.9712 27903152 PMC5562429

[B65] Houten S. M. Violante S. Ventura F. V. Wanders R. J. (2016). The Biochemistry and Physiology of Mitochondrial Fatty Acid β-Oxidation and its Genetic Disorders. Annu. Rev. Physiol. 78, 23–44. 10.1146/annurev-physiol-021115-105045 26474213

[B66] Hu A. Song B. L. (2019). The Interplay of Patched, Smoothened and Cholesterol in Hedgehog Signaling. Curr. Opin. Cel Biol 61, 31–38. 10.1016/j.ceb.2019.06.008 31369952

[B67] Huang G. M. Jiang Q. H. Cai C. Qu M. Shen W. (2015). SCD1 Negatively Regulates Autophagy-Induced Cell Death in Human Hepatocellular Carcinoma Through Inactivation of the AMPK Signaling Pathway. Cancer Lett. 358 (2), 180–190. 10.1016/j.canlet.2014.12.036 25528629

[B68] Ilic M. Ilic I. (2016). Epidemiology of Pancreatic Cancer. World J. Gastroenterol. 22 (44), 9694–9705. 10.3748/wjg.v22.i44.9694 27956793 PMC5124974

[B69] Imai H. Matsuoka M. Kumagai T. Sakamoto T. Koumura T. (2017). Lipid Peroxidation-dependent Cell Death Regulated by GPx4 and Ferroptosis. Curr. Top. Microbiol. Immunol. 403, 143–170. 10.1007/82_2016_508 28204974

[B70] Ito J. Komuro M. Parida I. S. Shimizu N. Kato S. Meguro Y. (2019). Evaluation of Lipid Oxidation Mechanisms in Beverages and Cosmetics via Analysis of Lipid Hydroperoxide Isomers. Sci. Rep. 9 (1), 7387. 10.1038/s41598-019-43645-1 31089240 PMC6517444

[B71] Ito J. Nakagawa K. Kato S. Hirokawa T. Kuwahara S. Nagai T. (2016). A Novel Chiral Stationary Phase HPLC-MS/MS Method to Discriminate Between Enzymatic Oxidation and Auto-Oxidation of Phosphatidylcholine. Anal. Bioanal. Chem. 408 (27), 7785–7793. 10.1007/s00216-016-9882-4 27549797

[B72] Iwamoto H. Abe M. Yang Y. Cui D. Seki T. Nakamura M. (2018). Cancer Lipid Metabolism Confers Antiangiogenic Drug Resistance. Cel Metab 28 (1), 104–117.e5. 10.1016/j.cmet.2018.05.005 29861385

[B73] Jiralerspong S. Goodwin P. J. (2016). Obesity and Breast Cancer Prognosis: Evidence, Challenges, and Opportunities. J. Clin. Oncol. 34 (35), 4203–4216. 10.1200/jco.2016.68.4480 27903149

[B74] Jun S. Y. Brown A. J. Chua N. K. Yoon J. Y. Lee J. J. Yang J. O. (2021). Reduction of Squalene Epoxidase by Cholesterol Accumulation Accelerates Colorectal Cancer Progression and Metastasis. Gastroenterology 160 (4), 1194–1207.e28. 10.1053/j.gastro.2020.09.009 32946903

[B75] Kagan V. E. Mao G. Qu F. Angeli J. P. Doll S. Croix C. S. (2017). Oxidized Arachidonic and Adrenic PEs Navigate Cells to Ferroptosis. Nat. Chem. Biol. 13 (1), 81–90. 10.1038/nchembio.2238 27842066 PMC5506843

[B76] Kajarabille N. Latunde-Dada G. O. (2019). Programmed Cell-Death by Ferroptosis: Antioxidants as Mitigators. Int. J. Mol. Sci. 20 (19), 4968. 10.3390/ijms20194968 PMC6801403 31597407

[B77] Kato S. Liberona M. F. Cerda-Infante J. Sánchez M. Henríquez J. Bizama C. (2018). Simvastatin Interferes with Cancer 'stem-Cell' Plasticity Reducing Metastasis in Ovarian Cancer. Endocr. Relat. Cancer 25 (10), 821–836. 10.1530/erc-18-0132 29848667

[B78] Kitajima S. Yoshida A. Kohno S. Li F. Suzuki S. Nagatani N. (2017). The RB-IL-6 axis Controls Self-Renewal and Endocrine Therapy Resistance by Fine-tuning Mitochondrial Activity. Oncogene 36 (36), 5145–5157. 10.1038/onc.2017.124 28481867

[B79] Kitson S. J. Rosser M. Fischer D. P. Marshall K. M. Clarke R. B. Crosbie E. J. (2019). Targeting Endometrial Cancer Stem Cell Activity with Metformin Is Inhibited by Patient-Derived Adipocyte-Secreted Factors. Cancers (Basel) 11 (5), 653. 10.3390/cancers11050653 PMC6562824 31083574

[B80] Kobayashi H. Enomoto A. Woods S. L. Burt A. D. Takahashi M. Worthley D. L. (2019). Cancer-associated Fibroblasts in Gastrointestinal Cancer. Nat. Rev. Gastroenterol. Hepatol. 16 (5), 282–295. 10.1038/s41575-019-0115-0 30778141

[B81] Koizume S. Miyagi Y. (2016). Lipid Droplets: A Key Cellular Organelle Associated with Cancer Cell Survival Under Normoxia and Hypoxia. Int. J. Mol. Sci. 17 (9), 1430. 10.3390/ijms17091430 PMC5037709 27589734

[B82] Koo J. H. Guan K. L. (2018). Interplay Between YAP/TAZ and Metabolism. Cel Metab 28 (2), 196–206. 10.1016/j.cmet.2018.07.010 30089241

[B83] Koohestanimobarhan S. Salami S. Imeni V. Mohammadi Z. Bayat O. (2018). Lipophilic Statins Antagonistically Alter the Major Epithelial‐to‐mesenchymal Transition Signaling Pathways in Breast Cancer Stem-like Cells via Inhibition of the Mevalonate Pathway. J. Cel Biochem 120, 2515–2531. 10.1002/jcb.27544 30191610

[B84] Kubo N. Araki K. Kuwano H. Shirabe K. (2016). Cancer-associated Fibroblasts in Hepatocellular Carcinoma. World J. Gastroenterol. 22 (30), 6841–6850. 10.3748/wjg.v22.i30.6841 27570421 PMC4974583

[B85] Kuramoto K. Yamamoto M. Suzuki S. Togashi K. Sanomachi T. Kitanaka C. (2021). Inhibition of the Lipid Droplet-Peroxisome Proliferator-Activated Receptor α Axis Suppresses Cancer Stem Cell Properties. Genes (Basel) 12 (1), 99. 10.3390/genes12010099 33466690 PMC7828779

[B86] Ladanyi A. Mukherjee A. Kenny H. A. Johnson A. Mitra A. K. Sundaresan S. (2018). Adipocyte-induced CD36 Expression Drives Ovarian Cancer Progression and Metastasis. Oncogene 37 (17), 2285–2301. 10.1038/s41388-017-0093-z 29398710 PMC5920730

[B87] Lee J. Y. Nam M. Son H. Y. Hyun K. Jang S. Y. Kim J. W. (2020). Polyunsaturated Fatty Acid Biosynthesis Pathway Determines Ferroptosis Sensitivity in Gastric Cancer. Proc. Natl. Acad. Sci. U S A. 117 (51), 32433–32442. 10.1073/pnas.2006828117 33288688 PMC7768719

[B88] Lee K. H. Lee M. S. Cha E. Y. Sul J. Y. Lee J. S. Kim J. S. (2017). Inhibitory Effect of Emodin on Fatty Acid Synthase, Colon Cancer Proliferation and Apoptosis. Mol. Med. Rep. 15 (4), 2163–2173. 10.3892/mmr.2017.6254 28260110 PMC5364834

[B89] Lei G. Zhang Y. Koppula P. Liu X. Zhang J. Lin S. H. (2020). The Role of Ferroptosis in Ionizing Radiation-Induced Cell Death and Tumor Suppression. Cell Res 30 (2), 146–162. 10.1038/s41422-019-0263-3 31949285 PMC7015061

[B90] Lewis C. A. Brault C. Peck B. Bensaad K. Griffiths B. Mitter R. (2015). SREBP Maintains Lipid Biosynthesis and Viability of Cancer Cells Under Lipid- and Oxygen-Deprived Conditions and Defines a Gene Signature Associated with Poor Survival in Glioblastoma Multiforme. Oncogene 34 (40), 5128–5140. 10.1038/onc.2014.439 25619842

[B91] Li D. Li Y. (2020). The Interaction Between Ferroptosis and Lipid Metabolism in Cancer. Signal. Transduct Target. Ther. 5 (1), 108. 10.1038/s41392-020-00216-5 32606298 PMC7327075

[B92] Li H. Feng Z. He M. L. (2020). Lipid Metabolism Alteration Contributes to and Maintains the Properties of Cancer Stem Cells. Theranostics 10 (16), 7053–7069. 10.7150/thno.41388 32641978 PMC7330842

[B93] Li J. Condello S. Thomes-Pepin J. Ma X. Xia Y. Hurley T. D. (2017a). Lipid Desaturation Is a Metabolic Marker and Therapeutic Target of Ovarian Cancer Stem Cells. Cell Stem Cell 20 (3), 303–314.e5. 10.1016/j.stem.2016.11.004 28041894 PMC5337165

[B94] Li L. Liu C. C. Chen X. Xu S. Hernandez Cortes-Manno S. Cheng S. H. (2017b). Mechanistic Study of Bakuchiol-Induced Anti-breast Cancer Stem Cell and In Vivo Anti-metastasis Effects. Front. Pharmacol. 8, 746. 10.3389/fphar.2017.00746 29093680 PMC5651275

[B95] Li P. Gong Z. Shultz L. D. Ren G. (2019). Mesenchymal Stem Cells: From Regeneration to Cancer. Pharmacol. Ther. 200, 42–54. 10.1016/j.pharmthera.2019.04.005 30998940 PMC6626571

[B96] Libby C. J. Tran A. N. Scott S. E. Griguer C. Hjelmeland A. B. (2018). The Pro-tumorigenic Effects of Metabolic Alterations in Glioblastoma Including Brain Tumor Initiating Cells. Biochim. Biophys. Acta Rev. Cancer 1869 (2), 175–188. 10.1016/j.bbcan.2018.01.004 29378228 PMC6596418

[B97] Liberti M. V. Locasale J. W. (2016). The Warburg Effect: How Does it Benefit Cancer Cells?. Trends Biochem. Sci. 41 (3), 211–218. 10.1016/j.tibs.2015.12.001 26778478 PMC4783224

[B98] Likus W. Siemianowicz K. Bieńk K. Pakuła M. Pathak H. Dutta C. (2016). Could Drugs Inhibiting the Mevalonate Pathway Also Target Cancer Stem Cells?. Drug Resist. Updat 25, 13–25. 10.1016/j.drup.2016.02.001 27155373

[B99] Ling B. Huang Z. Huang S. Qian L. Li G. Tang Q. (2021). Microenvironment Analysis of Prognosis and Molecular Signature of Immune-Related Genes in Lung Adenocarcinoma. Oncol. Res. 28 (6), 561–578. 10.3727/096504020x15907428281601 32471520 PMC7962936

[B100] Liu T. Jiang L. Tavana O. Gu W. (2019a). The Deubiquitylase OTUB1 Mediates Ferroptosis via Stabilization of SLC7A11. Cancer Res. 79 (8), 1913–1924. 10.1158/0008-5472.Can-18-3037 30709928 PMC6467774

[B101] Liu Y. Ren H. Zhou Y. Shang L. Zhang Y. Yang F. (2019b). The Hypoxia Conditioned Mesenchymal Stem Cells Promote Hepatocellular Carcinoma Progression Through YAP Mediated Lipogenesis Reprogramming. J. Exp. Clin. Cancer Res. 38 (1), 228. 10.1186/s13046-019-1219-7 31142342 PMC6540399

[B102] Lounis M. A. Péant B. Leclerc-Desaulniers K. Ganguli D. Daneault C. Ruiz M. (2020). Modulation of De Novo Lipogenesis Improves Response to Enzalutamide Treatment in Prostate Cancer. Cancers (Basel) 12 (11), 3339. 10.3390/cancers12113339 PMC7698241 33187317

[B103] Luanpitpong S. Janan M. Thumanu K. Poohadsuan J. Rodboon N. Klaihmon P. (2019). Deciphering the Elevated Lipid via CD36 in Mantle Cell Lymphoma with Bortezomib Resistance Using Synchrotron-Based Fourier Transform Infrared Spectroscopy of Single Cells. Cancers (Basel) 11 (4), 576. 10.3390/cancers11040576 PMC6521097 31022903

[B104] Ma X. L. Sun Y. F. Wang B. L. Shen M. N. Zhou Y. Chen J. W. (2019). Sphere-forming Culture Enriches Liver Cancer Stem Cells and Reveals Stearoyl-CoA Desaturase 1 as a Potential Therapeutic Target. BMC Cancer 19 (1), 760. 10.1186/s12885-019-5963-z 31370822 PMC6676608

[B105] Ma Y. Temkin S. M. Hawkridge A. M. Guo C. Wang W. Wang X. Y. (2018). Fatty Acid Oxidation: An Emerging Facet of Metabolic Transformation in Cancer. Cancer Lett. 435, 92–100. 10.1016/j.canlet.2018.08.006 30102953 PMC6240910

[B106] Magtanong L. Ko P. J. To M. Cao J. Y. Forcina G. C. Tarangelo A. (2019). Exogenous Monounsaturated Fatty Acids Promote a Ferroptosis-Resistant Cell State. Cell Chem Biol 26 (3), 420–432.e9. 10.1016/j.chembiol.2018.11.016 30686757 PMC6430697

[B107] Mai T. T. Hamaï A. Hienzsch A. Cañeque T. Müller S. Wicinski J. (2017). Salinomycin Kills Cancer Stem Cells by Sequestering Iron in Lysosomes. Nat. Chem. 9 (10), 1025–1033. 10.1038/nchem.2778 28937680 PMC5890907

[B108] Mancini R. Noto A. Pisanu M. E. De Vitis C. Maugeri-Saccà M. Ciliberto G. (2018). Metabolic Features of Cancer Stem Cells: The Emerging Role of Lipid Metabolism. Oncogene 37 (18), 2367–2378. 10.1038/s41388-018-0141-3 29445137

[B109] Mannes A. M. Seiler A. Bosello V. Maiorino M. Conrad M. (2011). Cysteine Mutant of Mammalian GPx4 Rescues Cell Death Induced by Disruption of the Wild-type Selenoenzyme. Faseb j 25 (7), 2135–2144. 10.1096/fj.10-177147 21402720

[B110] Martinez-Outschoorn U. E. Pestell R. G. Howell A. Tykocinski M. L. Nagajyothi F. Machado F. S. (2011). Energy Transfer in "parasitic" Cancer Metabolism: Mitochondria Are the Powerhouse and Achilles' Heel of Tumor Cells. Cell Cycle 10 (24), 4208–4216. 10.4161/cc.10.24.18487 22033146 PMC3272257

[B111] Mashima T. Seimiya H. Tsuruo T. (2009). De Novo fatty-acid Synthesis and Related Pathways as Molecular Targets for Cancer Therapy. Br. J. Cancer 100 (9), 1369–1372. 10.1038/sj.bjc.6605007 19352381 PMC2694429

[B112] Mason P. Liang B. Li L. Fremgen T. Murphy E. Quinn A. (2012). SCD1 Inhibition Causes Cancer Cell Death by Depleting Mono-Unsaturated Fatty Acids. PLoS One 7 (3), e33823. 10.1371/journal.pone.0033823 22457791 PMC3310881

[B113] Matrone A. Ferrari F. Santini F. Elisei R. (2020). Obesity as a Risk Factor for Thyroid Cancer. Curr. Opin. Endocrinol. Diabetes Obes. 27 (5), 358–363. 10.1097/med.0000000000000556 32740043

[B114] Mattoli L. Burico M. Fodaroni G. Tamimi S. Bedont S. Traldi P. (2016). New Frontiers in Pharmaceutical Analysis: A Metabolomic Approach to Check Batch Compliance of Complex Products Based on Natural Substances. J. Pharm. Biomed. Anal. 126, 156–162. 10.1016/j.jpba.2016.04.010 27155737

[B115] Menendez J. A. Lupu R. (2007). Fatty Acid Synthase and the Lipogenic Phenotype in Cancer Pathogenesis. Nat. Rev. Cancer 7 (10), 763–777. 10.1038/nrc2222 17882277

[B116] Menendez J. A. Mehmi I. Papadimitropoulou A. Vander Steen T. Cuyàs E. Verdura S. (2020). Fatty Acid Synthase Is a Key Enabler for Endocrine Resistance in Heregulin-Overexpressing Luminal B-like Breast Cancer. Int. J. Mol. Sci. 21 (20), 7661. 10.3390/ijms21207661 PMC7588883 33081219

[B117] Menendez J. A. Papadimitropoulou A. Vander Steen T. Cuyàs E. Oza-Gajera B. P. Verdura S. (2021). Fatty Acid Synthase Confers Tamoxifen Resistance to ER+/HER2+ Breast Cancer. Cancers 13 (5), 1132. 10.3390/cancers13051132 33800852 PMC7961649

[B118] Menter T. Tzankov A. Dirnhofer S. (2021). The Tumor Microenvironment of Lymphomas: Insights into the Potential Role and Modes of Actions of Checkpoint Inhibitors. Hematol. Oncol. 39 (1), 3–10. 10.1002/hon.2821 33105031

[B119] Mihaylova M. M. Cheng C. W. Cao A. Q. Tripathi S. Mana M. D. Bauer-Rowe K. E. (2018). Fasting Activates Fatty Acid Oxidation to Enhance Intestinal Stem Cell Function during Homeostasis and Aging. Cell Stem Cell 22 (5), 769–778.e4. 10.1016/j.stem.2018.04.001 29727683 PMC5940005

[B120] Mou Y. Wang J. Wu J. He D. Zhang C. Duan C. (2019). Ferroptosis, A New Form of Cell Death: Opportunities and Challenges in Cancer. J. Hematol. Oncol. 12 (1), 34. 10.1186/s13045-019-0720-y 30925886 PMC6441206

[B121] Mouhid L. Gómez de Cedrón M. García-Carrascosa E. Reglero G. Fornari T. Ramírez de Molina A. (2019). Yarrow Supercritical Extract Exerts Antitumoral Properties by Targeting Lipid Metabolism in Pancreatic Cancer. PLoS One 14 (3), e0214294. 10.1371/journal.pone.0214294 30913248 PMC6435158

[B122] Mukherjee A. Kenny H. A. Lengyel E. (2017). Unsaturated Fatty Acids Maintain Cancer Cell Stemness. Cell Stem Cell 20 (3), 291–292. 10.1016/j.stem.2017.02.008 28257705 PMC5956908

[B123] Najafi M. Farhood B. Mortezaee K. (2019). Extracellular Matrix (ECM) Stiffness and Degradation as Cancer Drivers. J. Cel Biochem 120 (3), 2782–2790. 10.1002/jcb.27681 30321449

[B124] Nimmakayala R. K. Leon F. Rachagani S. Rauth S. Nallasamy P. Marimuthu S. (2021). Metabolic Programming of Distinct Cancer Stem Cells Promotes Metastasis of Pancreatic Ductal Adenocarcinoma. Oncogene 40 (1), 215–231. 10.1038/s41388-020-01518-2 33110235 PMC10041665

[B125] Noto A. De Vitis C. Pisanu M. E. Roscilli G. Ricci G. Catizone A. (2017). Stearoyl-CoA-desaturase 1 Regulates Lung Cancer Stemness via Stabilization and Nuclear Localization of YAP/TAZ. Oncogene 36 (32), 4573–4584. 10.1038/onc.2017.75 28368399

[B126] Noto A. Raffa S. De Vitis C. Roscilli G. Malpicci D. Coluccia P. (2013). Stearoyl-CoA Desaturase-1 Is a Key Factor for Lung Cancer-Initiating Cells. Cel Death Dis 4 (12), e947. 10.1038/cddis.2013.444 PMC3877537 24309934

[B127] Okumura T. Ohuchida K. Sada M. Abe T. Endo S. Koikawa K. (2017). Extra-pancreatic Invasion Induces Lipolytic and Fibrotic Changes in the Adipose Microenvironment, with Released Fatty Acids Enhancing the Invasiveness of Pancreatic Cancer Cells. Oncotarget 8 (11), 18280–18295. 10.18632/oncotarget.15430 28407685 PMC5392327

[B128] Olzmann J. A. Carvalho P. (2019). Dynamics and Functions of Lipid Droplets. Nat. Rev. Mol. Cel Biol 20 (3), 137–155. 10.1038/s41580-018-0085-z PMC6746329 30523332

[B129] Ono M. Kosaka N. Tominaga N. Yoshioka Y. Takeshita F. Takahashi R. U. (2014). Exosomes from Bone Marrow Mesenchymal Stem Cells Contain a MicroRNA that Promotes Dormancy in Metastatic Breast Cancer Cells. Sci. Signal. 7 (332), ra63. 10.1126/scisignal.2005231 24985346

[B130] Orlando U. D. Castillo A. F. Medrano M. A. R. Solano A. R. Maloberti P. M. Podesta E. J. (2019). Acyl-CoA Synthetase-4 Is Implicated in Drug Resistance in Breast Cancer Cell Lines Involving the Regulation of Energy-dependent Transporter Expression. Biochem. Pharmacol. 159, 52–63. 10.1016/j.bcp.2018.11.005 30414939

[B131] Pacilli A. Calienni M. Margarucci S. D'Apolito M. Petillo O. Rocchi L. (2013). Carnitine-acyltransferase System Inhibition, Cancer Cell Death, and Prevention of Myc-Induced Lymphomagenesis. J. Natl. Cancer Inst. 105 (7), 489–498. 10.1093/jnci/djt030 23486551

[B132] Pan T. Liu J. Xu S. Yu Q. Wang H. Sun H. (2020). ANKRD22, A Novel Tumor Microenvironment-Induced Mitochondrial Protein Promotes Metabolic Reprogramming of Colorectal Cancer Cells. Theranostics 10 (2), 516–536. 10.7150/thno.37472 31903135 PMC6929986

[B133] Pandey P. R. Okuda H. Watabe M. Pai S. K. Liu W. Kobayashi A. (2011). Resveratrol Suppresses Growth of Cancer Stem-like Cells by Inhibiting Fatty Acid Synthase. Breast Cancer Res. Treat. 130 (2), 387–398. 10.1007/s10549-010-1300-6 21188630 PMC3404809

[B134] Park J. Morley T. S. Kim M. Clegg D. J. Scherer P. E. (2014). Obesity and Cancer-Mmechanisms Underlying Tumour Progression and Recurrence. Nat. Rev. Endocrinol. 10 (8), 455–465. 10.1038/nrendo.2014.94 24935119 PMC4374431

[B135] Pascual G. Avgustinova A. Mejetta S. Martín M. Castellanos A. Attolini C. S. (2017). Targeting Metastasis-Initiating Cells Through the Fatty Acid Receptor CD36. Nature 541 (7635), 41–45. 10.1038/nature20791 27974793

[B136] Peck B. Schug Z. T. Zhang Q. Dankworth B. Jones D. T. Smethurst E. (2016). Inhibition of Fatty Acid Desaturation Is Detrimental to Cancer Cell Survival in Metabolically Compromised Environments. Cancer Metab. 4, 6. 10.1186/s40170-016-0146-8 27042297 PMC4818530

[B137] Peck B. Schulze A. (2016). Lipid Desaturation - The Next Step in Targeting Lipogenesis in Cancer?. Febs j 283 (15), 2767–2778. 10.1111/febs.13681 26881388

[B138] Pei Y. Chen L. Huang Y. Wang J. Feng J. Xu M. (2019). Sequential Targeting TGF-β Signaling and KRAS Mutation Increases Therapeutic Efficacy in Pancreatic Cancer. Small 15 (24), e1900631. 10.1002/smll.201900631 31033217

[B139] Peiris-Pagès M. Martinez-Outschoorn U. E. Pestell R. G. Sotgia F. Lisanti M. P. (2016). Cancer Stem Cell Metabolism. Breast Cancer Res. 18 (1), 55. 10.1186/s13058-016-0712-6 27220421 PMC4879746

[B140] Peng G. Tang Z. Xiang Y. Chen W. (2019). Glutathione Peroxidase 4 Maintains A Stemness Phenotype, Oxidative Homeostasis and Regulates Biological Processes in Panc-1 C-ancer S-tem-like C-ells. Oncol. Rep. 41 (2), 1264–1274. 10.3892/or.2018.6905 30535490

[B141] Pinel A. Rigaudière J. P. Laillet B. Pouyet C. Malpuech-Brugère C. Prip-Buus C. (2016). N-3PUFA Differentially Modulate Palmitate-Induced Lipotoxicity Through Alterations of its Metabolism in C2C12 Muscle Cells. Biochim. Biophys. Acta 1861 (1), 12–20. 10.1016/j.bbalip.2015.10.003 26477381

[B142] Pinkham K. Park D. J. Hashemiaghdam A. Kirov A. B. Adam I. Rosiak K. (2019). Stearoyl CoA Desaturase Is Essential for Regulation of Endoplasmic Reticulum Homeostasis and Tumor Growth in Glioblastoma Cancer Stem Cells. Stem Cel Rep. 12 (4), 712–727. 10.1016/j.stemcr.2019.02.012 PMC6450460 30930246

[B143] Pisanu M. E. Maugeri-Saccà M. Fattore L. Bruschini S. De Vitis C. Tabbì E. (2018). Inhibition of Stearoyl-CoA Desaturase 1 Reverts BRAF and MEK Inhibition-Induced Selection of Cancer Stem Cells in BRAF-Mutated Melanoma. J. Exp. Clin. Cancer Res. 37 (1), 318. 10.1186/s13046-018-0989-7 30558661 PMC6298024

[B144] Pisanu M. E. Noto A. De Vitis C. Morrone S. Scognamiglio G. Botti G. (2017). Blockade of Stearoyl-CoA-Desaturase 1 Activity Reverts Resistance to Cisplatin in Lung Cancer Stem Cells. Cancer Lett. 406, 93–104. 10.1016/j.canlet.2017.07.027 28797843

[B145] Potze L. di Franco S. Kessler J. H. Stassi G. Medema J. P. (2016). Betulinic Acid Kills Colon Cancer Stem Cells. Curr. Stem Cel Res Ther 11 (5), 427–433. 10.2174/1574888x11666151203223512 26647913

[B146] Pouyafar A. Heydarabad M. Z. Abdolalizadeh J. Zade J. A. Rahbarghazi R. Talebi M. (2019). Modulation of Lipolysis and Glycolysis Pathways in Cancer Stem Cells Changed Multipotentiality and Differentiation Capacity Toward Endothelial Lineage. Cell Biosci 9, 30. 10.1186/s13578-019-0293-z 30962872 PMC6437852

[B147] Prasetyanti P. R. Medema J. P. (2017). Intra-tumor Heterogeneity from A Cancer Stem Cell Perspective. Mol. Cancer 16 (1), 41. 10.1186/s12943-017-0600-4 28209166 PMC5314464

[B148] Qu Q. Zeng F. Liu X. Wang Q. J. Deng F. (2016). Fatty Acid Oxidation and Carnitine Palmitoyltransferase I: Emerging Therapeutic Targets in Cancer. Cel Death Dis 7 (5), e2226. 10.1038/cddis.2016.132 PMC4917665 27195673

[B149] Quail D. F. Joyce J. A. (2013). Microenvironmental Regulation of Tumor Progression and Metastasis. Nat. Med. 19 (11), 1423–1437. 10.1038/nm.3394 24202395 PMC3954707

[B150] Quintana E. Shackleton M. Sabel M. S. Fullen D. R. Johnson T. M. Morrison S. J. (2008). Efficient Tumour Formation by Single Human Melanoma Cells. Nature 456 (7222), 593–598. 10.1038/nature07567 19052619 PMC2597380

[B151] Ricciardi M. R. Mirabilii S. Allegretti M. Licchetta R. Calarco A. Torrisi M. R. (2015). Targeting the Leukemia Cell Metabolism by the CPT1a Inhibition: Functional Preclinical Effects in Leukemias. Blood 126 (16), 1925–1929. 10.1182/blood-2014-12-617498 26276667

[B152] Rysman E. Brusselmans K. Scheys K. Timmermans L. Derua R. Munck S. (2010). De Novo lipogenesis Protects Cancer Cells from Free Radicals and Chemotherapeutics by Promoting Membrane Lipid Saturation. Cancer Res. 70 (20), 8117–8126. 10.1158/0008-5472.Can-09-3871 20876798

[B153] Saitta C. Pollicino T. Raimondo G. (2019). Obesity and Liver Cancer. Ann. Hepatol. 18 (6), 810–815. 10.1016/j.aohep.2019.07.004 31543467

[B154] Samudio I. Harmancey R. Fiegl M. Kantarjian H. Konopleva M. Korchin B. (2010). Pharmacologic Inhibition of Fatty Acid Oxidation Sensitizes Human Leukemia Cells to Apoptosis Induction. J. Clin. Invest. 120 (1), 142–156. 10.1172/jci38942 20038799 PMC2799198

[B155] Santos C. R. Schulze A. (2012). Lipid Metabolism in Cancer. Febs j 279 (15), 2610–2623. 10.1111/j.1742-4658.2012.08644.x 22621751

[B156] Seo Y. Kim J. Park S. J. Park J. J. Cheon J. H. Kim W. H. (2020). Metformin Suppresses Cancer Stem Cells Through AMPK Activation and Inhibition of Protein Prenylation of the Mevalonate Pathway in Colorectal Cancer. Cancers (Basel) 12 (9), 2554. 10.3390/cancers12092554 PMC7563617 32911743

[B157] Shao H. Mohamed E. M. Xu G. G. Waters M. Jing K. Ma Y. (2016). Carnitine Palmitoyltransferase 1A Functions to Repress FoxO Transcription Factors to Allow Cell Cycle Progression in Ovarian Cancer. Oncotarget 7 (4), 3832–3846. 10.18632/oncotarget.6757 26716645 PMC4826173

[B158] Shi J. Fu H. Jia Z. He K. Fu L. Wang W. (2016). High Expression of CPT1A Predicts Adverse Outcomes: A Potential Therapeutic Target for Acute Myeloid Leukemia. EBioMedicine 14, 55–64. 10.1016/j.ebiom.2016.11.025 27916548 PMC5161445

[B159] Shinohara H. Kumazaki M. Minami Y. Ito Y. Sugito N. Kuranaga Y. (2016). Perturbation of Energy Metabolism by Fatty-Acid Derivative AIC-47 and Imatinib in BCR-ABL-Harboring Leukemic Cells. Cancer Lett. 371 (1), 1–11. 10.1016/j.canlet.2015.11.020 26607903

[B160] Simeone P. Tacconi S. Longo S. Lanuti P. Bravaccini S. Pirini F. (2021). Expanding Roles of De Novo Lipogenesis in Breast Cancer. Int. J. Environ. Res. Public Health 18 (7), 3575. 10.3390/ijerph18073575 33808259 PMC8036647

[B161] Singh A. Ruiz C. Bhalla K. Haley J. A. Li Q. K. Acquaah-Mensah G. (2018). De Novo lipogenesis Represents a Therapeutic Target in Mutant Kras Non-small Cell Lung Cancer. Faseb j 32 (12), fj201800204. 10.1096/fj.201800204 PMC6219836 29906244

[B162] Singh S. R. Zeng X. Zhao J. Liu Y. Hou G. Liu H. (2016). The Lipolysis Pathway Sustains normal and Transformed Stem Cells in Adult Drosophila. Nature 538 (7623), 109–113. 10.1038/nature19788 27680705 PMC7798135

[B163] Snaebjornsson M. T. Janaki-Raman S. Schulze A. (2020). Greasing the Wheels of the Cancer Machine: The Role of Lipid Metabolism in Cancer. Cel Metab 31 (1), 62–76. 10.1016/j.cmet.2019.11.010 31813823

[B164] Song N. J. Yun U. J. Yang S. Wu C. Seo C. R. Gwon A. R. (2016). Notch1 Deficiency Decreases Hepatic Lipid Accumulation by Induction of Fatty Acid Oxidation. Sci. Rep. 6, 19377. 10.1038/srep19377 26786165 PMC4726366

[B165] Song Z. Xiang X. Li J. Deng J. Fang Z. Zhang L. (2020). Ruscogenin Induces Ferroptosis in Pancreatic Cancer Cells. Oncol. Rep. 43 (2), 516–524. 10.3892/or.2019.7425 31894321 PMC6967081

[B166] Stockwell B. R. Friedmann Angeli J. P. Bayir H. Bush A. I. Conrad M. Dixon S. J. (2017). Ferroptosis: A Regulated Cell Death Nexus Linking Metabolism, Redox Biology, and Disease. Cell 171 (2), 273–285. 10.1016/j.cell.2017.09.021 28985560 PMC5685180

[B167] Su L. J. Zhang J. H. Gomez H. Murugan R. Hong X. Xu D. (2019). Reactive Oxygen Species-Induced Lipid Peroxidation in Apoptosis, Autophagy, and Ferroptosis. Oxid Med. Cel Longev 2019, 5080843. 10.1155/2019/5080843 PMC6815535 31737171

[B168] Su R. Dong L. Li Y. Gao M. Han L. Wunderlich M. (2020). Targeting FTO Suppresses Cancer Stem Cell Maintenance and Immune Evasion. Cancer Cell 38 (1), 79–96.e11. 10.1016/j.ccell.2020.04.017 32531268 PMC7363590

[B169] Sun P. Xia S. Lal B. Shi X. Yang K. S. Watkins P. A. (2014). Lipid Metabolism Enzyme ACSVL3 Supports Glioblastoma Stem Cell Maintenance and Tumorigenicity. BMC Cancer 14, 401. 10.1186/1471-2407-14-401 24893952 PMC4055398

[B170] Takehara M. Sato Y. Kimura T. Noda K. Miyamoto H. Fujino Y. (2020). Cancer-associated Adipocytes Promote Pancreatic Cancer Progression Through SAA1 Expression. Cancer Sci. 111 (8), 2883–2894. 10.1111/cas.14527 32535957 PMC7419047

[B171] Terrén I. Orrantia A. Vitallé J. Zenarruzabeitia O. Borrego F. (2019). NK Cell Metabolism and Tumor Microenvironment. Front. Immunol. 10, 2278. 10.3389/fimmu.2019.02278 31616440 PMC6769035

[B172] Tesfay L. Paul B. T. Konstorum A. Deng Z. Cox A. O. Lee J. (2019). Stearoyl-CoA Desaturase 1 Protects Ovarian Cancer Cells from Ferroptotic Cell Death. Cancer Res. 79 (20), 5355–5366. 10.1158/0008-5472.Can-19-0369 31270077 PMC6801059

[B173] Tirinato L. Liberale C. Di Franco S. Candeloro P. Benfante A. La Rocca R. (2015). Lipid Droplets: A New Player in Colorectal Cancer Stem Cells Unveiled by Spectroscopic Imaging. Stem Cells 33 (1), 35–44. 10.1002/stem.1837 25186497 PMC4311668

[B174] Tirinato L. Pagliari F. Limongi T. Marini M. Falqui A. Seco J. (2017). An Overview of Lipid Droplets in Cancer and Cancer Stem Cells. Stem Cell Int 2017, 1656053. 10.1155/2017/1656053 PMC5572636 28883835

[B175] Tracz-Gaszewska Z. Dobrzyn P. (2019). Stearoyl-CoA Desaturase 1 as a Therapeutic Target for the Treatment of Cancer. Cancers (Basel) 11 (7), 948. 10.3390/cancers11070948 PMC6678606 31284458

[B176] Umeh-Garcia M. Simion C. Ho P. Y. Batra N. Berg A. L. Carraway K. L. (2020). A Novel Bioengineered miR-127 Prodrug Suppresses the Growth and Metastatic Potential of Triple-Negative Breast Cancer Cells. Cancer Res. 80 (3), 418–429. 10.1158/0008-5472.Can-19-0656 31694904 PMC7002233

[B177] Vancampfort D. Guelinckx H. De Hert M. Stubbs B. Soundy A. Rosenbaum S. (2014). Reliability and Clinical Correlates of the Astrand-Rhyming Sub-maximal Exercise Test in Patients with Schizophrenia or Schizoaffective Disorder. Psychiatry Res. 220 (3), 778–783. 10.1016/j.psychres.2014.08.049 25246409

[B178] Vanni S. Riccardi L. Palermo G. De Vivo M. (2019). Structure and Dynamics of the Acyl Chains in the Membrane Trafficking and Enzymatic Processing of Lipids. Acc. Chem. Res. 52 (11), 3087–3096. 10.1021/acs.accounts.9b00134 31364837

[B179] Vásquez-Bochm L. X. Velázquez-Paniagua M. Castro-Vázquez S. S. Guerrero-Rodríguez S. L. Mondragon-Peralta A. De La Fuente-Granada M. (2019). Transcriptome-based Identification of Lovastatin as a Breast Cancer Stem Cell-Targeting Drug. Pharmacol. Rep. 71 (3), 535–544. 10.1016/j.pharep.2019.02.011 31026757

[B180] Vazquez-Martin A. Corominas-Faja B. Cufi S. Vellon L. Oliveras-Ferraros C. Menendez O. J. (2013). The Mitochondrial H(+)-ATP Synthase and the Lipogenic Switch: New Core Components of Metabolic Reprogramming in Induced Pluripotent Stem (iPS) Cells. Cell Cycle 12 (2), 207–218. 10.4161/cc.23352 23287468 PMC3575450

[B181] Vergara D. Stanca E. Guerra F. Priore P. Gaballo A. Franck J. (2017). β-Catenin Knockdown Affects Mitochondrial Biogenesis and Lipid Metabolism in Breast Cancer Cells. Front. Physiol. 8, 544. 10.3389/fphys.2017.00544 28798698 PMC5529387

[B182] Vitale I. Manic G. Coussens L. M. Kroemer G. Galluzzi L. (2019). Macrophages and Metabolism in the Tumor Microenvironment. Cel Metab 30 (1), 36–50. 10.1016/j.cmet.2019.06.001 31269428

[B183] Vlashi E. Lagadec C. Vergnes L. Matsutani T. Masui K. Poulou M. (2011). Metabolic State of Glioma Stem Cells and Nontumorigenic Cells. Proc. Natl. Acad. Sci. U S A. 108 (38), 16062–16067. 10.1073/pnas.1106704108 21900605 PMC3179043

[B184] von Roemeling C. A. Marlow L. A. Wei J. J. Cooper S. J. Caulfield T. R. Wu K. (2013). Stearoyl-CoA Desaturase 1 Is a Novel Molecular Therapeutic Target for Clear Cell Renal Cell Carcinoma. Clin. Cancer Res. 19 (9), 2368–2380. 10.1158/1078-0432.Ccr-12-3249 23633458 PMC3644999

[B185] Vriens K. Christen S. Parik S. Broekaert D. Yoshinaga K. Talebi A. (2019). Evidence for an Alternative Fatty Acid Desaturation Pathway Increasing Cancer Plasticity. Nature 566 (7744), 403–406. 10.1038/s41586-019-0904-1 30728499 PMC6390935

[B186] Wahdan-Alaswad R. S. Cochrane D. R. Spoelstra N. S. Howe E. N. Edgerton S. M. Anderson S. M. (2014). Metformin-induced Killing of Triple-Negative Breast Cancer Cells Is Mediated by Reduction in Fatty Acid Synthase via miRNA-193b. Horm. Cancer 5 (6), 374–389. 10.1007/s12672-014-0188-8 25213330 PMC4570735

[B187] Walsh C. A. Akrap N. Garre E. Magnusson Y. Harrison H. Andersson D. (2020). The Mevalonate Precursor Enzyme HMGCS1 Is a Novel Marker and Key Mediator of Cancer Stem Cell Enrichment in Luminal and Basal Models of Breast Cancer. PLoS One 15 (7), e0236187. 10.1371/journal.pone.0236187 32692762 PMC7373278

[B188] Walther T. C. Chung J. Farese R. V. Jr. (2017). Lipid Droplet Biogenesis. Annu. Rev. Cel Dev Biol 33, 491–510. 10.1146/annurev-cellbio-100616-060608 PMC6986389 28793795

[B189] Wang B. Rong X. Palladino E. N. D. Wang J. Fogelman A. M. Martín M. G. (2018a). Phospholipid Remodeling and Cholesterol Availability Regulate Intestinal Stemness and Tumorigenesis. Cell Stem Cell 22 (2), 206–220.e4. 10.1016/j.stem.2017.12.017 29395055 PMC5807072

[B190] Wang C. Shao L. Pan C. Ye J. Ding Z. Wu J. (2019a). Elevated Level of Mitochondrial Reactive Oxygen Species via Fatty Acid β-oxidation in Cancer Stem Cells Promotes Cancer Metastasis by Inducing Epithelial-Mesenchymal Transition. Stem Cel Res Ther 10 (1), 175. 10.1186/s13287-019-1265-2 PMC6567550 31196164

[B191] Wang C. Xu H. Deng J. Yu H. Chen Y. Wang S. (2019b). Prognostic Factors in Pediatric Pneumococcal Meningitis Patients in Mainland China: A Retrospective Multicenter Study. Infect. Drug Resist. 12, 1501–1512. 10.2147/idr.S193671 31239727 PMC6560191

[B192] Wang G. Xu J. Zhao J. Yin W. Liu D. Chen W. (2020a). Arf1-mediated Lipid Metabolism Sustains Cancer Cells and its Ablation Induces Anti-tumor Immune Responses in Mice. Nat. Commun. 11 (1), 220. 10.1038/s41467-019-14046-9 31924786 PMC6954189

[B193] Wang J. Li Y. (2019). CD36 Tango in Cancer: Signaling Pathways and Functions. Theranostics 9 (17), 4893–4908. 10.7150/thno.36037 31410189 PMC6691380

[B194] Wang S. Li Y. Xing C. Ding C. Zhang H. Chen L. (2020b). Tumor Microenvironment in Chemoresistance, Metastasis and Immunotherapy of Pancreatic Cancer. Am. J. Cancer Res. 10 (7), 1937–1953. 32774994 PMC7407356

[B195] Wang T. Fahrmann J. F. Lee H. Li Y. J. Tripathi S. C. Yue C. (2018b). JAK/STAT3-Regulated Fatty Acid β-Oxidation Is Critical for Breast Cancer Stem Cell Self-Renewal and Chemoresistance. Cel Metab 27 (1), 1357. 10.1016/j.cmet.2017.11.00110.1016/j.cmet.2018.04.018 PMC6116734 29874570

[B196] Wang X. Huang Z. Wu Q. Prager B. C. Mack S. C. Yang K. (2017). MYC-regulated Mevalonate Metabolism Maintains Brain Tumor-Initiating Cells. Cancer Res. 77 (18), 4947–4960. 10.1158/0008-5472.Can-17-0114 28729418 PMC5600855

[B197] Wang X. Sun Y. Wong J. Conklin D. S. (2013). PPARγ Maintains ERBB2-Positive Breast Cancer Stem Cells. Oncogene 32 (49), 5512–5521. 10.1038/onc.2013.217 23770845 PMC3898098

[B198] Wang Y. Zhao G. Condello S. Huang H. Cardenas H. Tanner E. J. (2021). Frizzled-7 Identifies Platinum-Tolerant Ovarian Cancer Cells Susceptible to Ferroptosis. Cancer Res. 81 (2), 384–399. 10.1158/0008-5472.CAN-20-1488 33172933 PMC7855035

[B199] Wang Z. Wang N. Liu P. Xie X. (2016). AMPK and Cancer. Exp. Suppl. 107, 203–226. 10.1007/978-3-319-43589-3_9 27812982

[B200] Wen Y. A. Xiong X. Zaytseva Y. Y. Napier D. L. Vallee E. Li A. T. (2018). Downregulation of SREBP Inhibits Tumor Growth and Initiation by Altering Cellular Metabolism in colon Cancer. Cel Death Dis 9 (3), 265. 10.1038/s41419-018-0330-6 PMC5833501 29449559

[B201] Wohlhieter C. A. Richards A. L. Uddin F. Hulton C. H. Quintanal-Villalonga À. Martin A. (2020). Concurrent Mutations in STK11 and KEAP1 Promote Ferroptosis Protection and SCD1 Dependence in Lung Cancer. Cel Rep 33 (9), 108444. 10.1016/j.celrep.2020.108444 PMC7722473 33264619

[B202] Wu H. Liu B. Chen Z. Li G. Zhang Z. (2020a). MSC-induced lncRNA HCP5 Drove Fatty Acid Oxidation Through miR-3619-5p/AMPK/PGC1α/CEBPB Axis to Promote Stemness and Chemo-Resistance of Gastric Cancer. Cel Death Dis 11 (4), 233. 10.1038/s41419-020-2426-z PMC7162922 32300102

[B203] Wu L. Lin Q. Ma Z. Chowdhury F. A. Mazumder M. H. H. Du W. (2020b). Mesenchymal PGD2 Activates an ILC2-Treg Axis to Promote Proliferation of Normal and Malignant HSPCs. Leukemia 34 (11), 3028–3041. 10.1038/s41375-020-0843-8 32366935 PMC7606225

[B204] Wu Q. Li B. Li Z. Li J. Sun S. Sun S. (2019a). Cancer-associated Adipocytes: Key Players in Breast Cancer Progression. J. Hematol. Oncol. 12 (1), 95. 10.1186/s13045-019-0778-6 31500658 PMC6734503

[B205] Wu Q. Zhou L. Lv D. Zhu X. Tang H. (2019b). Exosome-mediated Communication in the Tumor Microenvironment Contributes to Hepatocellular Carcinoma Development and Progression. J. Hematol. Oncol. 12 (1), 53. 10.1186/s13045-019-0739-0 31142326 PMC6542024

[B206] Xu T. Ding W. Ji X. Ao X. Liu Y. Yu W. (2019). Molecular Mechanisms of Ferroptosis and its Role in Cancer Therapy. J. Cel Mol Med 23 (8), 4900–4912. 10.1111/jcmm.14511 PMC6653007 31232522

[B207] Xu X. Zhang X. Wei C. Zheng D. Lu X. Yang Y. (2020). Targeting SLC7A11 Specifically Suppresses the Progression of Colorectal Cancer Stem Cells via Inducing Ferroptosis. Eur. J. Pharm. Sci. 152, 105450. 10.1016/j.ejps.2020.105450 32621966

[B208] Yamada N. Karasawa T. Kimura H. Watanabe S. Komada T. Kamata R. (2020). Ferroptosis Driven by Radical Oxidation of N-6 Polyunsaturated Fatty Acids Mediates Acetaminophen-Induced Acute Liver Failure. Cel Death Dis 11 (2), 144. 10.1038/s41419-020-2334-2 PMC7039960 32094346

[B209] Yang D. Peng M. Hou Y. Qin Y. Wan X. Zhu P. (2020a). Oxidized ATM Promotes Breast Cancer Stem Cell Enrichment Through Energy Metabolism Reprogram-Mediated Acetyl-CoA Accumulation. Cel Death Dis 11 (7), 508. 10.1038/s41419-020-2714-7 PMC7343870 32641713

[B210] Yang W. S. Stockwell B. R. (2016). Ferroptosis: Death by Lipid Peroxidation. Trends Cel Biol 26 (3), 165–176. 10.1016/j.tcb.2015.10.014 PMC4764384 26653790

[B211] Yang Y. Li X. Wang T. Guo Q. Xi T. Zheng L. (2020b). Emerging Agents That Target Signaling Pathways in Cancer Stem Cells. J. Hematol. Oncol. 13 (1), 60. 10.1186/s13045-020-00901-6 32456660 PMC7249421

[B212] Yao J. Man S. Dong H. Yang L. Ma L. Gao W. (2018). Combinatorial Treatment of Rhizoma Paridis Saponins and Sorafenib Overcomes the Intolerance of Sorafenib. J. Steroid Biochem. Mol. Biol. 183, 159–166. 10.1016/j.jsbmb.2018.06.010 29932973

[B213] Yasumoto Y. Miyazaki H. Vaidyan L. K. Kagawa Y. Ebrahimi M. Yamamoto Y. (2016). Inhibition of Fatty Acid Synthase Decreases Expression of Stemness Markers in Glioma Stem Cells. PLoS One 11 (1), e0147717. 10.1371/journal.pone.0147717 26808816 PMC4726602

[B214] Ye H. Adane B. Khan N. Sullivan T. Minhajuddin M. Gasparetto M. (2016). Leukemic Stem Cells Evade Chemotherapy by Metabolic Adaptation to an Adipose Tissue Niche. Cell Stem Cell 19 (1), 23–37. 10.1016/j.stem.2016.06.001 27374788 PMC4938766

[B215] Yi M. Li J. Chen S. Cai J. Ban Y. Peng Q. (2018). Emerging Role of Lipid Metabolism Alterations in Cancer Stem Cells. J. Exp. Clin. Cancer Res. 37 (1), 118. 10.1186/s13046-018-0784-5 29907133 PMC6003041

[B216] Yu T. Wang Y. Fan Y. Fang N. Wang T. Xu T. (2019). CircRNAs in Cancer Metabolism: A Review. J. Hematol. Oncol. 12 (1), 90. 10.1186/s13045-019-0776-8 31484561 PMC6727394

[B217] Yu Y. Kim H. Choi S. Yu J. Lee J. Y. Lee H. (2021). Targeting A Lipid Desaturation Enzyme, SCD1, Selectively Eliminates Colon Cancer Stem Cells Through the Suppression of Wnt and NOTCH Signaling. Cells 10 (1), 106. 10.3390/cells10010106 33430034 PMC7826607

[B218] Yuan J. Dong X. Yap J. Hu J. (2020). The MAPK and AMPK Signalings: Interplay and Implication in Targeted Cancer Therapy. J. Hematol. Oncol. 13 (1), 113. 10.1186/s13045-020-00949-4 32807225 PMC7433213

[B219] Zechner R. Zimmermann R. Eichmann T. O. Kohlwein S. D. Haemmerle G. Lass A. (2012). FAT SIGNALS-Llipases and Lipolysis in Lipid Metabolism and Signaling. Cel Metab 15 (3), 279–291. 10.1016/j.cmet.2011.12.018 PMC3314979 22405066

[B220] Zeng F. Yao M. Wang Y. Zheng W. Liu S. Hou Z. (2021). Fatty Acid β-oxidation Promotes Breast Cancer Stemness and Metastasis via the miRNA-328-3p-Cpt1a Pathway. Cancer Gene Ther. 10.1038/s41417-021-00348-y PMC8940624 34045663

[B221] Zhang B. Bie Q. Wu P. Zhang J. You B. Shi H. (2018a). PGD2/PTGDR2 Signaling Restricts the Self-Renewal and Tumorigenesis of Gastric Cancer. Stem Cells 36 (7), 990–1003. 10.1002/stem.2821 29604141

[B222] Zhang H. Deng T. Liu R. Ning T. Yang H. Liu D. (2020). CAF Secreted miR-522 Suppresses Ferroptosis and Promotes Acquired Chemo-Resistance in Gastric Cancer. Mol. Cancer 19 (1), 43. 10.1186/s12943-020-01168-8 32106859 PMC7045485

[B223] Zhang H. Li H. Ho N. Li D. Li S. (2012). Scd1 Plays a Tumor-Suppressive Role in Survival of Leukemia Stem Cells and the Development of Chronic Myeloid Leukemia. Mol. Cel Biol 32 (10), 1776–1787. 10.1128/MCB.05672-11 PMC3347408 22431519

[B224] Zhang L. Ge L. Parimoo S. Stenn K. Prouty S. M. (1999). Human Stearoyl-CoA Desaturase: Alternative Transcripts Generated from a Single Gene by Usage of Tandem Polyadenylation Sites. Biochem. J. 340 ( Pt 1) (Pt 1Pt 1), 255–264. 10.1042/bj3400255 10229681 PMC1220244

[B225] Zhang L. Pan Y. Qin G. Chen L. Chatterjee T. K. Weintraub N. L. (2014). Inhibition of Stearoyl-coA Desaturase Selectively Eliminates Tumorigenic Nanog-Positive Cells: Improving the Safety of iPS Cell Transplantation to Myocardium. Cell Cycle 13 (5), 762–771. 10.4161/cc.27677 24394703 PMC3979912

[B226] Zhang X. Du L. Qiao Y. Zhang X. Zheng W. Wu Q. (2019a). Ferroptosis Is Governed by Differential Regulation of Transcription in Liver Cancer. Redox Biol. 24, 101211. 10.1016/j.redox.2019.101211 31108460 PMC6526247

[B227] Zhang X. Hu F. Li G. Li G. Yang X. Liu L. (2018b). Human Colorectal Cancer-Derived Mesenchymal Stem Cells Promote Colorectal Cancer Progression Through IL-6/JAK2/STAT3 Signaling. Cel Death Dis 9 (2), 25. 10.1038/s41419-017-0176-3 PMC5833830 29348540

[B228] Zhang Y. Liu Y. Duan J. Wang H. Zhang Y. Qiao K. (2019b). Cholesterol Depletion Sensitizes Gallbladder Cancer to Cisplatin by Impairing DNA Damage Response. Cell Cycle 18 (23), 3337–3350. 10.1080/15384101.2019.1676581 31599189 PMC6927696

[B229] Zhao C. Wu M. Zeng N. Xiong M. Hu W. Lv W. (2020). Cancer-associated Adipocytes: Emerging Supporters in Breast Cancer. J. Exp. Clin. Cancer Res. 39 (1), 156. 10.1186/s13046-020-01666-z 32787888 PMC7425140

[B230] Zhao Y. Zhao W. Lim Y. C. Liu T. (2019). Salinomycin-Loaded Gold Nanoparticles for Treating Cancer Stem Cells by Ferroptosis-Induced Cell Death. Mol. Pharm. 16 (6), 2532–2539. 10.1021/acs.molpharmaceut.9b00132 31009228

